# Aryl hydrocarbon receptor facilitates HSV-1 lytic infection by enhancing viral gene transcription and receptor expression

**DOI:** 10.3389/fcimb.2025.1548038

**Published:** 2025-06-25

**Authors:** Pu Huang, Xiaohong Pan, Hongli Chen, Mengyue Lei, Ying Ma, Xiaomei Guo, Jiaxin Xie, Jixiong Li, Jing Sun, Yunzhang Hu, Jiandong Shi

**Affiliations:** ^1^ Yunnan Provincial Key Laboratory of Vector-borne Diseases Control and Research, Institute of Medical Biology, Chinese Academy of Medical Sciences and Peking Union Medical College, Kunming, China; ^2^ Honghe Health Vocational College, Honghe, China; ^3^ Kunming Medical University, Kunming, China; ^4^ National Kunming High-level Biosafety Primate Research Center, Institute of Medical Biology, Chinese Academy of Medical Sciences and Peking Union Medical College, Kunming, China

**Keywords:** HSV-1, lytic infection, AhR, host factor, antiviral target

## Abstract

Herpes simplex virus type 1 (HSV-1) is a major human pathogen with significant morbidity in neonates and immunocompromised individuals. However, antiviral drugs targeting HSV-1 are emerging with antiviral resistance, highlighting the need to identify new targets for future treatment. Once HSV-1 enters the host cells, it recruits host factors to facilitate viral life cycle. In this study, we showed that aryl hydrocarbon receptor (AhR), a ligand-activated nuclear receptor, is required for HSV-1 effective replication and offers an opportunity for pharmacological intervention. Our results showed that HSV-1 infection activates AhR signaling in an interferon-dependent manner. Pharmacological inhibition or knockout of AhR reduced the expression of viral proteins and infectious progeny, while increased AhR signaling promoted the expression of viral proteins and viral replication. Mechanistically, AhR formed a transcription complex with cyclin T1, VP16 and RNA Pol II in the nucleus, bound to viral gene promoters, and promoted their transcription. Additionally, AhR promoted viral replication partially by facilitating the expression of multiple viral receptors. Collectively, AhR is a proviral host factor for HSV-1, and thus may be used as a promising host-directed antiviral target.

## 1 Introduction

Herpes simplex virus type I (HSV-1) is among the most ubiquitous human infections that belongs to the alpha herpesvirus.Worldwide, ~90% of people have HSV-1 or HSV-2, or both viruses and increased morbidity and mortality have been observed in neonates and patients with immunodeficiency (Zhu and Viejo-Borbolla, 2021). The pathogenesis of HSV-1 has not been fully understood, especially the key cellular and molecular events of viral lytic infection. During initial exposure, HSV-1 attacks mucosal epithelial cells, including epidermal keratinocytes, which are also the primary portal of entry and spread through the epithelium (Atyeo et al., 2021). HSV-1 is a neurotropic herpesvirus that establishes latency within sensory neurons, spreading to the peripheral sensory neurons, such as the trigeminal ganglia and the central nervous system (CNS), establishing lifelong latent infection (Roizman and Sears, 1987). HSV-1 can periodically reactivate after stress or other similar stimuli, transport to the skin via axons of neurons, and re-enter the lytic infection cycle (Cliffe and Wilson, 2017), triggering new infections. Multiple viral and cellular factors are involved in HSV replication during lytic infection. Once inside the host cell, the virus utilizes a series of cellular proteins to promote its lifecycle. Lytic infection by HSV-1 triggers cellular responses as the virus strives to express its own genes and replicate. HSV-1 genes are expressed in three distinct stages and cascaded, immediate early (IE), early (E), and late (L). The IE proteins, including ICP0, ICP4, ICP22 and ICP27, play key roles in regulating the expression of the E and L genes during infection (Zhu and Jones, 2018; Honess and Roizman, 1974). After host infection with HSV-1, the viral tegument protein VP16 released into host cells forms complexes with host cytokine 1 (HCF-1) and octameric-binding protein-1 (Oct-1), which binds to the promoter of the *IE* genes and drives their expression (Johnson et al., 1999; Wysocka and Herr, 2003). More than ten known *E* gene products participate in the replication of the viral genome and promote the expression of the *L* genes (Shapiro et al., 2021; Rajcáni and Durmanová, 2000).

The viral receptors play an important role in the intercellular transmission of viruses. The entry of HSV-1 daughter virus into new target cells is a highly regulated process, and membrane fusion is a critical step. Membrane fusion is catalyzed by gB, a member of the class III fusion protein family (Khadr et al., 2019). However, activation of gB occurs only under appropriate conditions, and to initiate the process, gD must firstly bind to one of the host cell receptors of nectin-1, herpesvirus entry receptor (HVEM), or 3-O-sulfated HS (Connelly et al., 2021). Interactions between gB and paired immunoglobulin-like type 2 receptor α (PILRA), myelin-associated glycoproteins, and non-muscle myosin IIAs have also been demonstrated to be involved in HSV-1 entry into cells (Satoh et al., 2008; Arii et al., 2010). The mice lacking HVEM and nectin-1 receptors exhibit enhanced resistance to HSV challenge (Kopp et al., 2013; Taylor et al., 2007). Thus, the expression level of the viral receptors directly affects the intercellular infection efficiency of the virus.

Drugs against herpesvirus infection in the clinic are mainly of two types: nucleoside analogs such as acyclovir targeting DNA polymerase/thymidine kinase and helicase-primase inhibitors such as amenamevir (ASP2151), with both types having the ability to reduce or stop the replication of the viral genome. Despite the effectiveness of these drugs in controlling viral infections, a potential concern is that these agents, when activated, may also target the host’s DNA polymerase and lead to higher toxicity (Sadowski et al., 2021). The viral resistance to acyclovir (ACV), currently the mainstay for HSV-1, has been reported with higher rates in immunocompromised patients. The emergence of virus resistance to antiviral drugsemphasizes the need to identify new targets for future antiviral therapy. Thus, identifying key host factors that regulate viral infection and pathogenicity is an important prerequisite for promoting diagnosis and therapeutic strategies.

This study demonstrated that aryl hydrocarbon receptor (AhR), a ligand-driven nuclear receptor, is required for HSV-1 replication and offers an opportunity for pharmacological intervention. AhR is a ligand-activated transcription factor that acts as a nuclear receptor for environmental pollutants polycyclic aromatic hydrocarbons (PAHs), mediating exogenous chemical or physical stimuli (O’driscoll et al., 2018). When unactivated, AhR is widely expressed in the cytoplasm of a variety of cells. Kynurenine (Kyn) is an endogenous natural ligand of AhR, which can induce and activate AhR signaling. Once activated, AhR enters the nucleus and binds to the AhR nuclear transposon (ARNT) to form a dimer, specifically binding to the AhR reaction element and inducing the expression of a series of downstream genes, including cytochrome enzyme 4501A1 (cytochrome P4501A1 and CYP1A1) (Kukhanova et al., 2014). Beyond its canonical function in detoxifying xenobiotics (e.g., polycyclic aromatic hydrocarbons, dioxins), AhR is activated by endogenous ligands, including metabolites derived from tryptophan catabolism, such as kynurenine (Kyn). AhR activation is intricately linked to tryptophan metabolism through the kynurenine pathway. Tryptophan is catabolized by IDO1 (induced by inflammatory cytokines like IFN-γ) or TDO2 (constitutively active in the liver) to generate Kyn, a potent endogenous AhR ligand. This pathway serves as a critical interface between immune responses and metabolic reprogramming. Recent studies have also confirmed that AhR regulates viral infection and immunity, thereby providing a survival advantage to many viruses. For example, tryptophan metabolism activates the AhR-mediated pathway to promote HIV-1 infection and reactivation (Ahmad and Wilson, 2020). The AhR-P4501A1 pathway controls lipid accumulation and enhances hepatitis C virus assembly (Hilterbrand and Heldwein, 2019). Zika virus infection activates AhR and limits the production of interferon type I (IFN-I) (Owen et al., 2015). AhR activation disrupts the activation of influenza virus-specific CD8+ T cells in the lungs (Heming et al., 2017). Our recent study has also reported that AhR promotes SARS-CoV-2 replication by inhibiting type I interferon and promoting the expression of host cell receptor ACE2 (Shi et al., 2023). Additionally, our recent study has also reported that the addition of tryptophan metabolite kynurenine (a natural ligand for AhR) promoted HSV-1 replication^25^. Overall, these studies suggest that AhR, as an immune regulatory molecule, is involved in the regulation of various viral infections and host immune responses.

In the present study, we reported that HSV-1 infection induces the production of kynurenine (Kyn), which in turn activates AhR and then enters the nucleus and forms a transcription complex with cyclin T1, VP16, and RNAPII, which binds to promoters of HSV-1 immediate early genes *ICP0* and *ICP27*. The subregion positively regulates the transcription of HSV-1 immediate early genes, thereby promoting the replication of HSV-1. We also found that activating AhR could promote the expression of HSV-1 receptors, thereby promoting HSV-1 infection. The IFN-IDO-Kyn-AhR axis represents a vulnerability for HSV-1. Pharmacological inhibition of AhR (e.g., CH223191) or blockade of Kyn production (e.g., IDO1 inhibitors) could disrupt this axis, offering a host-directed strategy to limit viral replication without directly targeting viral enzymes. Overall, these findings collectively define AhR as a proviral host factor for HSV-1 and identify AhR antagonists as candidate therapeutics for managing HSV-1 infection.

## 2 Materials and methods

### 2.1 Cells and viruses

Multiple cell lines, including human lung fibroblasts KMB17 (from Institute of Medical Biology, Chinese Academy of Medical Sciences), human hepatoma cell line Huh7 (Wuhan Pricella Biotechnology Co., Ltd.), human lung cancer cell line A549 (Wuhan Pricella Biotechnology Co., Ltd.), human bronchial epithelial cells line BEAS-2B (Wuhan Pricella Biotechnology Co., Ltd.), Vero cells (from the ATCC, CCL-81), and human neuroblastoma cells SK-N-SH (Wuhan Pricella Biotechnology Co., Ltd.) were used in this study. The AhR-deficient HepG2 cells (AhR^−/−^ HepG2) was generated by CRISPR/Cas9-mediated genome engineering (HanBio, Shanghai, China) as described in our previous study (Shi et al., 2023). These cell lines were cultured in Dulbecco’s Modified Eagle Medium (DMEM) (C3113-0500, VivacellCertified) supplemented with a 10% Fetal Bovine Serum (FBS) (C04001-050X10, VivacellCertified) and 1% penicillin and streptomycin in a humidified atmosphere containing 5%CO_2_/95% air at 37°C (Stockinger et al., 2014). The HSV-1 virus strain 17 (GenBank number: NC_001806.2) was amplified and titrated in Vero cells.

### 2.2 Extraction of genomic DNA from HSV-1 virus

Viral genome DNA is extracted by the AxyPrep Body Fluid Viral DNA/RNA Miniprep Kit (AP-MN-BF-VNA-250, Axygen) following the manufacturer’s protocol.

### 2.3 Plasmid construction and siRNA transfection

The plasmids containing the ICP0, ICP27, or TK promoter sequences, named pGL3-Enhancer-ICP0, pGL3-Enhancer-ICP27, and pGL3-Enhancer-TK, were constructed. Briefly, the viral gene promoters were amplified using specific primers and inserted into the pGL3-Enhance vector using KpnI and XhoI enzyme sites. Standard plasmids for absolute quantitative PCR were also constructed by amplifying viral genomic DNA fragments; amplification products were ligated into the T vector (pClone007 Versatile Simple Vector Kit, Qiagen) and confirmed by sequencing. The primers for the ICP0, ICP27, and TK promoter sequences and the primers for the HSV-1 standard plasmid are listed in **Supplementary Table S1**. The plasmid PCDNA3.1-AhR used for overexpressing AhR was constructed (Beijing Yibaike Biotechnology Co.,Ltd). SiRNA targeting AhR was also transfected according to the manufacturer’s instructions (Cata. No. SR319302, OriGene Technologies).

### 2.4 RNA extraction, reverse transcription, and real-time PCR

Total RNA was extracted from cells using TRIzol (Invitrogen) and reverse-transcribed into cDNA using the GoScript reverse transcription system (A5001, Promega). To detect the copy number of HSV-1 genomic DNA in mouse organs, absolute quantitative PCR was used. Based on the Ct value of the unknown sample, the copy number of the sample can be obtained in the standard curve. In addition, real-time PCR was performed in a relatively quantitative manner, and the real-time PCR data of the target genes were normalized to the expression of the reference gene GAPDH to calculate relative gene expression (Chu et al., 2022). All PCR amplification was performed using the GoTaq^®^ qPCR Master Mix (A6001, Promega) on the CFX-96 Real-Time RT-PCR Detection System (Bio-Rad). Sequences of all quantitative PCR primers are listed in **Supplementary Table S2**.

### 2.5 ELISA measurement of interferon, tryptophan, and kynurenines in the cell supernatant

The content of interferon in the supernatant of the culture was measured by the human IFN-beta ELISA Kit (KE00187, Proteintech). The content of tryptophan in the supernatant was measured using the Human Tryptophane ELISA Kit (DEIA074Y, Creative Diagnostics), and the content of canine uric acid in the supernatant was measured using the L-Kyrene ELISA Kit (DEIA097J, Creative Diagnostics). All operations are carried out according to the instructions provided by the kit manufacturer.

### 2.6 Immunofluorescence

The cells were seeded in six-well plates with 1.5 × 10^6^ cells per well and cultured overnight. The original medium was removed the following day and incubated with serum-free medium and the HSV-1 virus (multiplicity of infection (MOI) = 0.1) for 1 h. Then, the medium was removed, the cells were washed twice with phosphate-buffered saline (PBS), and a maintenance solution (with 2% FBS) was added to continue the culture. After 24 h, the cells were collected, fixed with 4% paraformaldehyde for 30 min, permeabilized with 0.5% Triton X-100 at room temperature for 20 min, and blocked with 5% bovine serum albumin (BSA) for 30 min. Then, each slide was incubated with diluted primary antibody in a humidified chamber box overnight at 4°C. The following antibodies were used: anti-ICP5 (1:500, Ab6508, Abcam), anti-AhR (1:500, GTX129013, GeneTex), anti-Cyclin T1 (1:200, #81464, Cell Signaling), anti-RNA Pol II (2ug/ml, 91151, Active Motif), anti-VP16 (1:500, ab110226, Abcam), anti-Cyclin T1 (1.5μg/ml, sc-271348, SANTA CRuZ BIOTECHNOLOGY). Subsequently, the slides were washed with PBST and incubated with FITC-labeled (1:200, SA00003-1, Proteintech) or Cy3-labeled (1:100, SA00009-2, Proteintech) fluorescent secondary antibody at 37°C for 1 h. Finally, the slides were sealed by dropping the sealing liquid containing DAPI and an anti-fluorescence quencher, and the images were observed and collected using a panoramic MIDI digital scanner (3D HISTECH, Budapest, Hungary), as previously described (Leiva et al., 2021).

### 2.7 Nucleoplasmic separation

The SK-N-SH cells were infected with HSV-1 (MOI=0.1), with DMSO as the negative control and Kyn (211007, CD) and I3S (#BCCF7082, SIGMA) as the positive control. The nucleus and cytoplasm were separated by the NE-PER Nuclear and Cytoplasmic Extraction Reagents Kit (78833, Thermo) after 24 hours, and the samples after separation were used for immunoblotting to detect the amount of protein in the cytoplasm and nucleus.

### 2.8 Immunoblotting

Cells from one confluent well of a six-well plate were lysed by adding RIPA lysis buffer supplemented with a protease inhibitor cocktail in an ice bath for 30 min. The lysates were centrifuged at 12,000× g for 10 min at 4°C, and the supernatants containing total proteins were prepared. Then, 6 × loading buffer was added into the supernatant and placed in a 95°C water bath for denaturation for 10 min. Moreover, the denatured total cell lysates were separated by 4–20% BisTris SDS-PAGE and transferred to PVDF membranes (Millipore). The membranes were blocked in 5% non-fat dry milk and inoculated with diluted primary antibody overnight at 4°C. The membranes were washed four times with TBS + 0.1%Tween-20. The membranes were then incubated with horseradish peroxidase (HRP)-conjugated secondary antibodies (goat anti-mouse, Cat. No. SA00001-1, Proteintech; goat anti-rabbit, Cat. No. SA00001-2, Proteintech) and developed using enhanced chemiluminescence (ECL) substrate. The results were confirmed by at least three biological replicates.

The following antibodies were used for immunoblotting: anti-ICP5 (1:2000, Ab6508, Abcam), anti-AhR (1:2000, GTX129013, GeneTex), anti-Lamin b1 (1:10000, 66095-1-Lg, Proteintech), anti-GAPDH (1:50000, 60004-1-Ig, Proteintech), anti-ICP0 (1:5000, Ab6513, Abcam), anti-ICP27 (1:500, Sc-69806, SANTA CRuZ BIOTECHNOLOGY), anti-TK (1:1000, Ls-c415730, LifeSpan Biosciences), anti-RNA Pol II (1:1000, Ab5131, Abcam), anti-Cyclin T1 (1:2000, #81464, Cell Signaling), anti-MYH9 (1:10000, 11128-1-AP, Proteintech), anti-MAG (1:2000, 14386-1-AP, Proteintech), anti-PVRL1/nectin1 (1:2000, 24713-1-AP, Proteintech), anti-PILRA (1:300, 11818-1-AP, Proteintech), anti-TNFRSF14/HVEM (1:1000, 10138-1-AP, Proteintech).

### 2.9 Determination of 50% cell culture infectious dose

Vero cells were evenly mixed with fresh DMEM containing 4% FBS and seeded in a 96-well plate with about 5 × 10^4^ cells per well. Then, the collected infectious supernatant was diluted ten-fold in fresh serum-free DMEM medium and added to the 96 well plates seeded with Vero cells for incubation for 5 days. Each dilution was tested with eight replicates for each experiment, and the wells were observed and scored for the presence or absence of cytopathic effect (CPE). Then, 50% cell culture infectious dose (CCID50) values were calculated using the Karber method (Lei et al., 2021).

### 2.10 Dual-luciferase reporter assay

The activity of the *Firefly* and *Ranilla* luciferase was measured using the Dual-Luciferase Reporter Assay System Technical Manual kit (E1910, Promega). The relative luciferase activity was calculated as described in the manufacturer’s instructions. The data were collected by FlexStation 3 Multi-Mode Microplate Reader.

### 2.11 Chromatin immunoprecipitation

The cell lysate of HepG2 cells infected with HSV for 12 hours was used for chromatin immunoprecipitation analysis by using ChIP-IT^®^Express Enzymatic Kits (53009, Active Motif) as described in the kit, simply preparing chromatin fragments by digestion, followed by anti-AhR (#83200, Cell Signaling) and anti-IgG (#5414, Cell Signaling) antibody co-precipitated with the treated chromatin fragment, eluted, uncrosslinked and used as a template for qRT-PCR to detect AhR-bound fragments on the promoter regions of ICP0 and ICP27. Sequences of all ChIP primers are listed in **Supplementary Table S3**.

### 2.12 Co-immunoprecipitation

HepG2 cells were infected with HSV-1 (MOI=10). After 6 hours, the protein was collected, and Co-IP was performed using the Immunoprecision Kit with Protein A+G Magnetic Beads kit (P2179S, Beyotime). The specific steps were performed as follows: (1) binding of anti Cyclin T1 (# 81464, Cell Signaling), anti AhR (# 83200, Cell Signaling), anti RNA Pol II (91151, Active Motiv), and anti IgG (# 5414, Cell Signaling) antibodies to Protein A+G, and prepare the antibodies with a final concentration of 25 μg/ml; (2) collected cell lysate was co incubated with the antibody protein A+G complex for immunoprecipitation; (3) the protein was washed and subjected to Western blotting to detect the co binding of RNAP-II, AhR, CyclinT1, and VP16 proteins.

### 2.13 Cell viability assay

Cells cultured in 96 well plates (5×10^4^ cells per well) were exposed to three concentration gradients of CH223191 (3858, TOCRIS) or I3S(#BCCF7082, SIGMA) for 24 h with 5 wells per concentration. Then, cell viability was measured with the CellTiter AQueous One Solution Cell Proliferation Assay (G3582, Promega, USA), according to the manufacturer’s instructions. The absorption rate at 490nm wavelength of each well was measured on a SpectraMax i3 multimode microplate reader at 490 nm. (Molecular Device).

### 2.14 Transfection of plasmids and siRNA

Lipofectamine™3000 kit (L3000015, Thermo Fisher Scientific) was used to transfect cells that reached 70–90% confluence. Briefly, Lipofectamine™ 3000 reagent and plasmid were diluted (1:1 ratio) using Opti-MEM™ medium (31985-070, Gibco) and then mixed with P3000™ reagent at room temperature for 10–15 min. Finally, plasmid-lipid complexes were then added to the cells. When transfecting siRNA, the same protocol (see above) was applied, except for the P3000™ reagent, which was not used. Ordinary plasmids were transfected with adherent cells at an amount of 2.5 μg per well; after preliminary exploration, it was determined that the amount of plasmid with PGL3-Enhancer as the carrier in the 24-well plate was 50 ng/well, and the amount of PRL-CMV plasmid was 5 ng/well. The final concentration of siRNA transfection of adherent cells was 10 nM.

### 2.15 Statistical analysis

All experiments were performed in triplicate. All statistical analyses were performed using the GraphPad Prism (Versio 9.0.0) software. The difference between the two sets of data was analyzed using a two-tailed unpaired Student’s t-test. The P value < 0.05 represented a statistically significant difference.

## 3 Results

### 3.1 AhR signaling is activated by HSV-1 in an IFN-IDO-Kyn-dependent pathway

**Figure 1:**
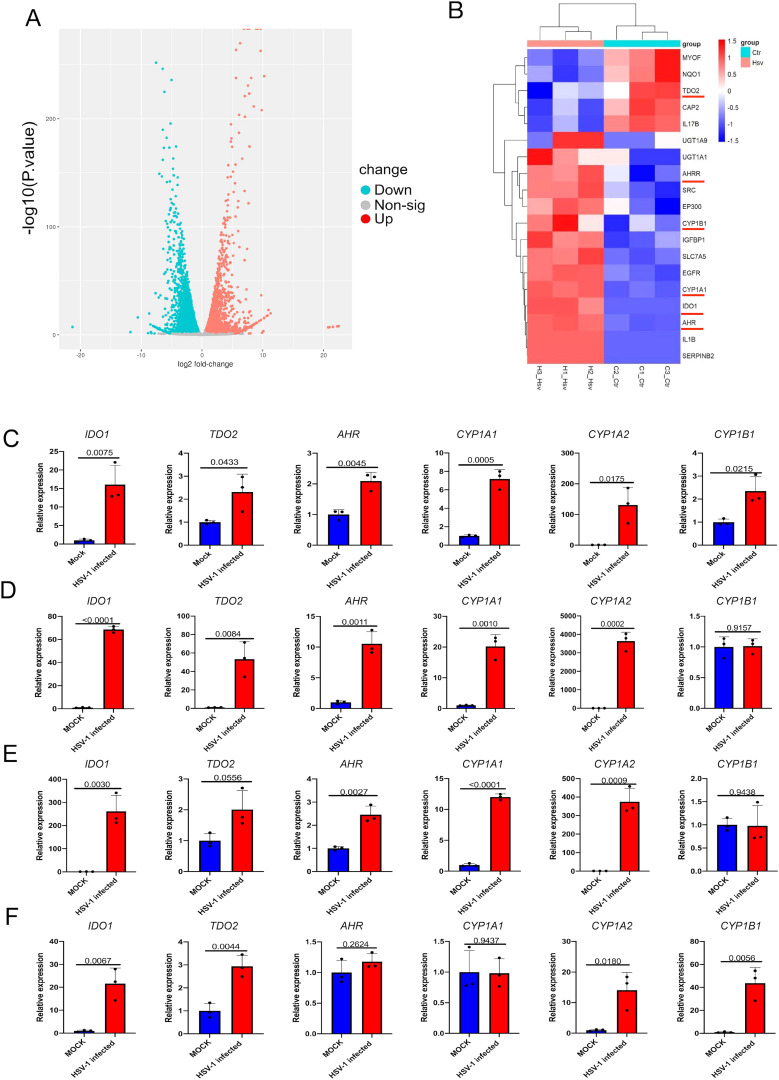
Infection with HSV-1 activates the AhR signaling. **(A)** Volcano map of DEGs after HSV-1 infection with KMB17 cells. The RNA sequencing data from our previous study (GSE103763) (Shi et al., 2018). **(B)** Clustering heat map of the genes in AhR signaling in HSV-1 infected and mock-infected KMB17 cells. **(C-F)** The relative expression of AhR signaling genes after HSV-1 infection of KMB17 **(C)**, BEAS-2B **(D)**, A549 **(E)**, and HepG2 **(F)** cells by relative quantitative PCR compared with the control group (n=3, MOI=0.01, 48hpi). Data from at least three independent experiments (mean ± SD). P-values were determined using a two-tailed, unpaired Student’s t-test.

To investigate host factors involved in HSV-1 lytic infection, we analyzed the RNA sequencing data sets (GSE103763) from HSV-1 infected and mock-infected KMB17 cells in our previous study (Shi et al., 2018). Cluster analysis of differentially expressed genes (DEGs) revealed that the multiple genes in AhR signaling pathways (*IDO1, TDO2, AhR, CYP1A1, CYP1A2, CYP1B1*) were significantly upregulated (**Figures 1A, B**) in HSV-1 infected cells compared with mock-infected cells. Similar expression changes of these DEGs have also been confirmed by qRT-PCR in HSV-1-infected and mock-infected KMB17 cells (**Figure 1C**). To analyze whether the activation of AhR signaling by HSV-1 is a universal characteristic, multiple types of cell lines, including the BEAS-2B, A549, and HepG2 cells, were also infected with HSV-1, and the relative expression of related genes in the AhR signaling pathway were measured. The results showed that upon HSV-1 infection, most genes in the AhR signaling pathway were significantly upregulated (**Figures 1D–F**), suggesting that HSV-1 universally activated the AhR signaling in multiple cell lines.

**Figure 2:**
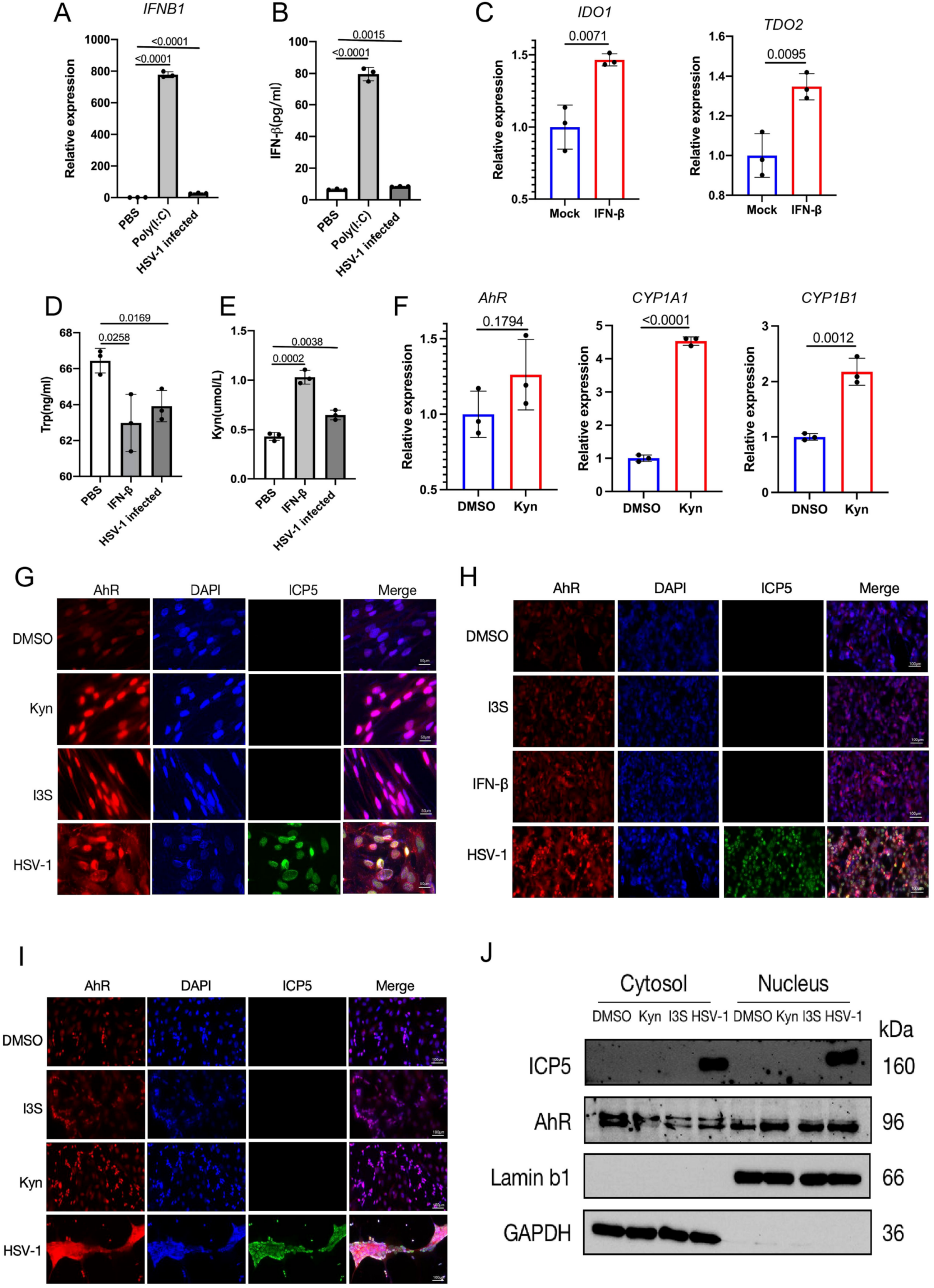
HSV-1 infection activates the AhR signaling in an interferon-dependent manner. **(A, B)** KMB17 cells were infected with HSV-1 (MOI=0.01, 48hpi, n=3); PBS was the negative control, Poly(I:C) was the positive control, and the relative expression of β-interferon (IFNB1) in the cells was detected by qPCR **(A)**. The content of β-interferon in culture supernatant was detected by ELISA **(B)**. **(C)** KMB17 cells were treated with 10 ng/ml β-interferon or DMSO (control group). The relative expression of *IDO1* and *TDO2* genes was detected after 24 h. **(D, E)** KMB17 cells were infected with HSV-1 (MOI=0.01, 48hpi, n=3) or PBS (negative control) or interferon (positive control). The contents of tryptophan **(D)** and kynuremine **(E)** in the culture supernatant were detected by ELISA. **(F)** KMB17 cells were treated with KYN (400 μM) or DMSO (control group). The relative expression of *AHR*, *CYP1A1*, and *CYP1B1* genes was detected after 48 h. Data from at least three independent experiments (mean ± SD). P-values were determined using a two-tailed, unpaired Student’s t-test. **(G-J)** HSV-1 infection promotes the entry of AhR into the nucleus. **(G-I)** The DMSO-treated group was used as negative control and I3S or Kyn or IFN-β-treated groups were used as positive control. KMB17 **(G)**, HepG2 **(H)**, and SK-N-SH cells **(I)** were infected with HSV-1 (MOI=1); immunofluorescence was performed after 24 h. The red fluorescence represents the AhR protein, the blue fluorescence represents the nucleus, and the green fluorescence represents viral ICP5 protein. Scale bar, 50 μm. **(J)** SK-N-SH cells were infected with HSV-1 (MOI=0.1); the DMSO-treated group was used as a negative control, and Kyn or I3S-treated groups were used as a positive control. Nucleoplasmic separation was performed after 24 h, followed by Western blot assay to detect the content of AhR protein in cytoplasm and nucleus.

Next, we investigated how AhR signaling is activated by HSV-1 infection *in vitro*. We noticed that the relative expression of the *IFNB1* gene and the content of β-interferon in cell culture supernatant was slightly upregulated in HSV-1 infected KMB17 cells (**Figures 2A, B**). HSV-1 infection stimulates interferon production; however, the relatively low IFN induction levels observed in infected cells compared to positive controls may be related to viral antagonism of the host’s innate immune defense mechanisms. Moreover, the β-Interferon upregulated the relative expression of *IDO1* and *TDO2* genes, key rate-limiting enzymes for tryptophan metabolism to kynurenine (**Figure 2C**). More importantly, the tryptophan content in the supernatant of HSV-1 infected cells was significantly reduced, and the downstream metabolite of the tryptophan, kynurenine content, was significantly increased (**Figures 2D, E**). The resulting kynurenine acted as a natural ligand for AhR to activate the expression of AhR’s downstream genes *CYP1A1 and CYP1B1* (**Figure 2F**). Overall, these results suggested that HSV-1 slightly induces cellular IFN-β, thereby promoting tryptophan to kynurenine metabolism and activating the AhR signaling.

As AhR is a nuclear receptor, its activation is mainly characterized by nuclear translocation after being driven by ligands. Thus, to further observe whether AhR undergoes nuclear translocation after HSV-1 infection, the immunofluorescence and immunoblotting were performed in different cell lines infected with HSV-1. These results showed that AhR fluorescence intensity within the nucleus was significantly enhanced in KMB17, HepG2 and SK-N-SH cells that treated with Kyn, IFN-β I3S or HSV-1 compared with DMSO-treated control groups (**Figures 2G–I**). In addition, we also performed nucleoplasmic isolation experiments in SK-N-SH cells to measure the content of AhR protein in the cytoplasm and nucleus after HSV-1 infection using western blotting. The results showed that the content of AhR protein in the cytoplasm in those groups treated with Kyn, I3S or HSV-1 was significantly reduced compared with the DMSO-treated group. Yet, the content of AhR protein in the nucleus in those groups treated with Kyn, I3S or HSV-1was significantly increased compared with the DMSO-treated group (**Figure 2J**). Taken together, these results indicated that HSV-1 infection activates the AhR signaling and promotes its nuclear translocation in an IFN-IDO-Kyn-dependent pathway. Significantly, we also observed that SARS-CoV-2 infection activates AhR signaling in an IFN-IDO-Kyn-dependent pathway in our previous study (Shi et al., 2023). Therefore, activation of AhR signaling by viral infection may be a common phenomenon in the interaction between viruses and host cells.

### 3.2 AhR signaling boosts HSV-1 replication *in vitro*

**Figure 3:**
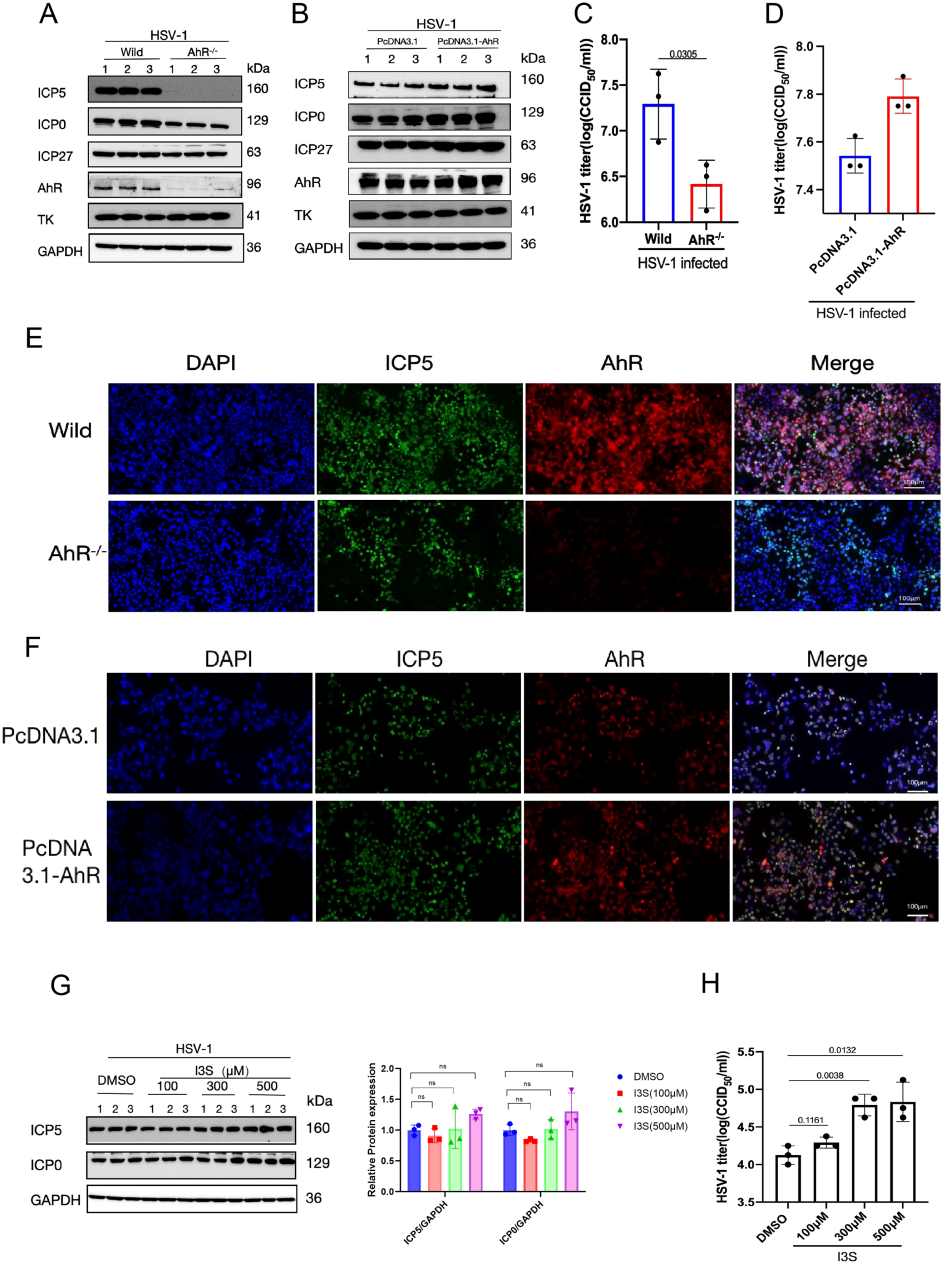
AhR signaling boosts HSV-1 replication. **(A)** Wild-type and AhR^-/-^ HepG2 cells were infected with HSV-1 (MOI=1), and protein was harvested at 12 hpi. **(B)** HepG2 cells were transfected with pcDNA3.1-AhR or pcDNA 3.1 plasmids, followed by infection by HSV-1 (MOI=0.1). The proteins were harvested at 24 hpi. For **(A, B)**, a Western blot was conducted to detect the expression levels of viral proteins ICP5, ICP0, ICP27, TK, and host proteins AhR; GAPDH was used as a loading control. **(C)** HepG2 cells were infected with HSV-1 (MOI=0.1), and the cell supernatant was collected at 48 hpi for CCID50 assay. **(D)** HepG2 cells transfected with pcDNA3.1-AhR or pcDNA 3.1 plasmids were infected with HSV-1 (MOI=0.1), and the cell supernatant was collected at 48 hpi for CCID50 assay. **(E)** Wild-type and AhR^-/-^ HepG2 cells were infected with HSV-1 (MOI=1), and immunofluorescence was performed at 24 hpi. **(F)** HepG2 cells transfected with pcDNA3.1-AhR or pcDNA 3.1 plasmids were infected with HSV-1 (MOI=1), and immunofluorescence was performed at 24 hpi. The red fluorescence represents the AhR protein, the blue fluorescence represents the nucleus, and the green fluorescence represents the ICP5 protein. Scale bar, 100 μm. **(G)** Vero cells were pretreated with AhR agonists I3S at different concentrations (100 μM, 300 μM, and 500 μM) for 24h. After infection with HSV-1 (MOI=1) for 12h, the protein was collected and Western blot was performed to detect the expression of ICP5, ICP0 and GAPDH proteins. Band intensities for ICP0, ICP5, and GAPDH (loading control) from Western blot experiments were quantified using ImageJ software. Relative protein levels were calculated as the ratio of ICP0 or ICP5 signal to GAPDH. One-way ANOVA with Dunnett’s *post-hoc* test (vs. DMSO control) was performed. In the picture,ns represents no significant difference. **(H)** At 24 hpi, cell supernatant was collected to determine virus titer by CCID50 assay. Data from at least three independent experiments (mean ± SD). P-values were determined using a two-tailed, unpaired Student’s t-test.

To investigate the effect of AhR on HSV-1 replication, we detected the expression of multiple viral proteins ICP5, ICP0, ICP27 and TK in wild-type and AhR knockout HepG2 cells (AhR^-/-^ HepG2) infected with HSV-1 by Western blot and immunofluorometric assay. The results showed that the expression of the immediate early proteins ICP0, ICP27 and late protein ICP5 of HSV-1 decreased significantly in HepG2 AhR^-/-^ cells compared with wild-type HepG2 cells (**Figure 3A**). The titer of HSV-1 is also significantly reduced in AhR^-/-^ HepG2 cells compared with wild-type HepG2 cells (**Figure 3C**), as determined by the CCID50 assay. Immunofluorescence analysis of the ICP5 protein further showed that the fluorescence of the ICP5 protein in AhR^-/-^ HepG2 cells is significantly weakened compared with wild-type HepG2 cells (**Figure 3E**).

To further determine the effect of AhR signaling on HSV-1 replication, the AhR was overexpressed in HSV-1 infeced cells by transfecting plasmid pcDNA3.1-AhR. The expression of viral proteins and viral titer was measured by Western blot and CCID50 assay. As expected, the expression levels of viral proteins ICP0, ICP27, and ICP5 increased significantly in HepG2 cells overexpressed AhR compared with cells transfected with empty vector (**Figure 3B**). The fluorescence signal of viral proteins in HepG2 cells overexpressed AhR was significantly enhanced (**Figure 3F**) compared with cells transfected with empty vector; and viral titer was also slightly increased (**Figure 3D**). Yet, we did not observe changes in viral Thymidine kinase (TK) expression in AhR deficient or overexpressed HepG2 cells, suggesting that AhR has no regulatory effect on *TK* gene.

To further analyze the effects of AhR signaling for HSV-1 replication, the AhR agonists I3S with different concentrations was added to Vero cells and followed by infecting with HSV-1. The results showed that the expression levels of viral proteins ICP0 and ICP5 were slightly increased in I3S-treated cells compared with DMSO-treated controls (**Figure 3G**). Immunofluorescence analysis of the ICP5 protein showed significantly enhanced fluorescence signal in I3S-treated cells compared with DMSO-treated cells (**Supplementary Figure S1**). Consistent with this trend, viral titers were markedly elevated at higher I3S concentrations (300 and 500 μM; **Figure 3H**). Thus, AhR inhibition or knockdown revealed that the AhR supports viral replication, significantly increasing the yield of infectious progeny. Taken together, these results indicated that AhR signaling boosts HSV-1 replication and acts as a host proviral factor, thereby deeming AhR as a potential antiviral therapeutic target.

### 3.3 Pharmacological inhibition of AhR suppresses HSV-1 replication *in vitro*

**Figure 4:**
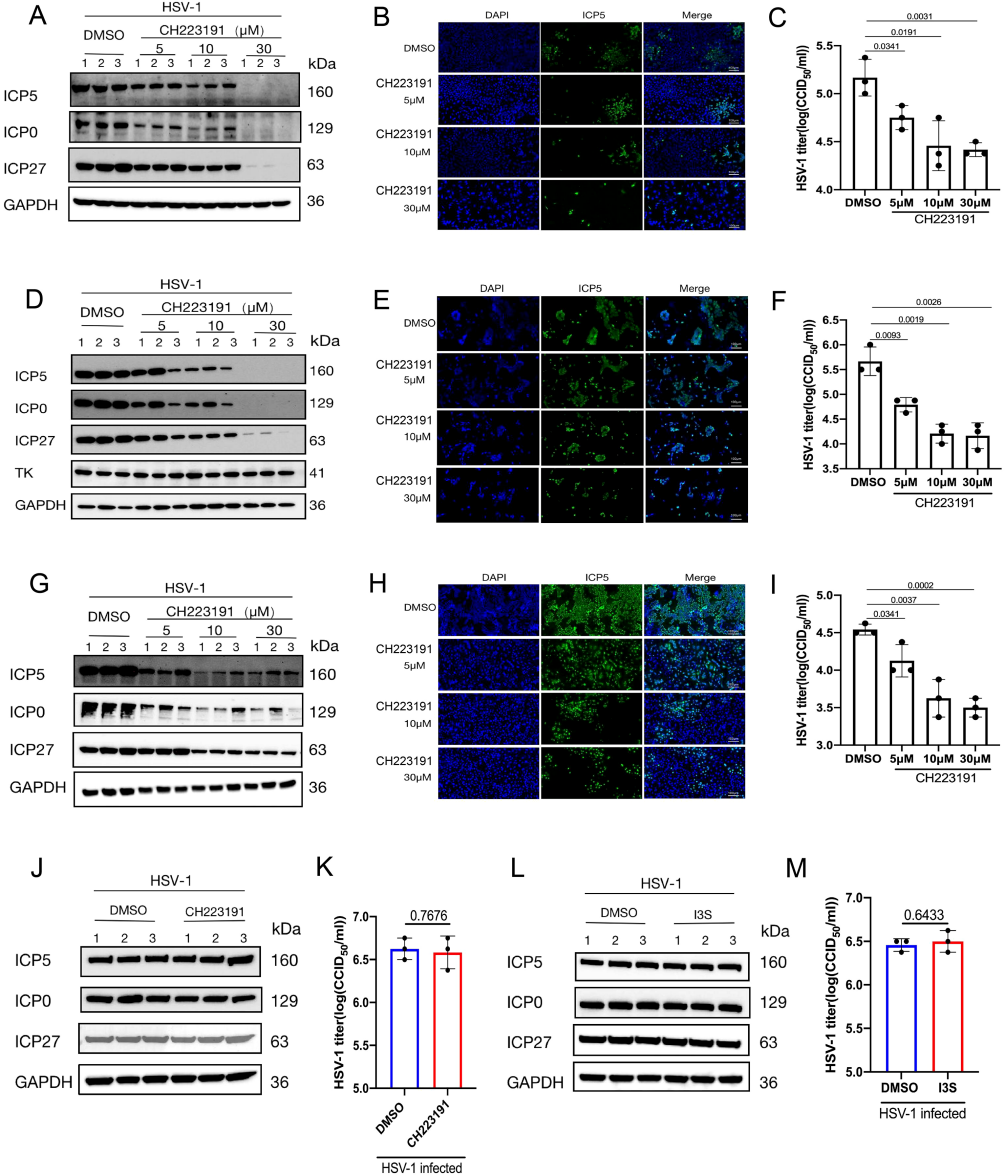
Pharmacological inhibition of AhR suppresses HSV-1 replication *in vitro*. **(A, D, G)** Huh7 **(A)**, SK-N-SH **(D)** and Vero cells **(G)** were pretreated with CH223191 at different concentrations (5μM, 10μM, and 30μM) for 24h and then infected with HSV-1 (MOI=0.1). At 24 hpi. The expression of ICP5, ICP0, ICP27 and GAPDH proteins were detected by Western blot. **(B, E, H)** Huh7 **(B)**, SK-N-SH **(E)**, and Vero cells **(H)** were pretreated with CH223191 at different concentrations (5μM, 10μM, 30μM) for 24h, and then infected with HSV-1 (MOI=1) for 24h, and then used for immunofluorescence. Viral ICP5 protein was observed under a fluorescence microscope. The blue fluorescence represents nuclear; the green fluorescence represents ICP5 protein. Scale bar, 100 μm. **(C, F, I)** Huh7 **(C)**, SK-N-SH **(F)** and Vero cells **(I)** were pretreated with CH223191 at different concentrations (5μM, 10μM, and 30μM) for 24h and then treated with with HSV-1 (MOI=0.01) for 24h. Then, viral titer in cell supernatant was determined by CCID_50_ assay. **(J, K)** AhR^-/-^ HepG2 cells were pretreated with 500 μM CH223191 for 24h. At 24hpi after HSV-1 infection (MOI=1), the protein was collected for measuring the expression of ICP5, ICP0, ICP27 and GAPDH proteins by Western blot **(J)**. Viral titer **(K)** was determined by CCID50 assay with cell supernatant. **(L, M)** AhR^-/-^ HepG2 were pretreated with 50μM I3S for 24h, and the protein was collected for measuring the expression of ICP5, ICP0, ICP27 and GAPDH proteins by Western blot **(L)**. **(M)** Viral titer in cell supernatant was determined by CCID50 assay. Data from at least three independent experiments (mean ± SD). P-values were determined using a two-tailed, unpaired Student’s t-test.

Since AhR is essential for HSV-1 effective replication, we next evaluated the effect of pharmacological inhibition of AhR on the HSV-1 replication. The viral proteinand viral titerwere analyzed in threecell lines infected with HSV-1. Briefly, AhR antagonist CH223191 with different concentrations were added to Huh7, SK-N-SH or Vero cells infected with HSV-1. At 24 hpi, the expression of viral protein was detected by Western blot and Immunofluorometric assay, and the virus titer was determined by CCID_50_ assay. The results showed that the expression of viral proteins ICP5, ICP0, and ICP27 significantly decreased with the increase of CH223191 concentration compared with the DMSO-treated groups in Huh7 cells (**Figure 4A**), SK-N-SH cells (**Figure 4D**), and Vero cells (**Figure 4G**). Furthermore, as the concentration of CH223191 increased, the fluorescence intensity of the ICP5 protein gradually became lower in Huh7 cells (**Figure 4B**), SK-N-SH cells (**Figure 4E**), and Vero cells (**Figure 4H**). Consistent with these results, viral titer in the cell culture supernatant was significantly decreased in Huh7 cells (**Figure 4C**), SK-N-SH cells (**Figure 4F**), and Vero cells (**Figure 4I**). Remarkably, in Huh7 cells and SK-N-SH cells, almost no protein bands were detected for the viral ICP5 and ICP0 when the CH223191 concentration reached at 30 μM. Taken together, these results demonstrated that the pharmacological inhibition of AhR by antagonist CH223191 can suppress the replication of HSV-1 *in vitro*.

Next, in order to determine the effects of I3S and CH223191 on HSV-1 replication by AhR signaling, the AhR signaling-deficient AhR^-/-^ HepG2 cells were added I3S or CH223191 and followd by infecting HSV-1. At 24 hpi, viral ICP5, ICP0 and ICP27 proteins were measured by Western blot.The viral titer was detected by CCID_50_ assay. As expected, no significant changes were observed in viral ICP5, ICP0 and ICP27 proteins expression (**Figure 4J**) and viral titer (**Figure 4K**) between CH223191 treated AhR^-/-^ HepG2 cells and DMSO-treated AhR^-/-^ HepG2 cells. Similar results were also observed in AhR^-/-^ HepG2 cells treated with I3S or DMSO (**Figures 4L, M**). Notably, the specific regulatory effect of I3S or CH223191 on AhR signaling has been confirmed in our previous study (Shi et al., 2023). No cytotoxicity was observed for CH223191-and I3S-treated cells within the concentration range used in this study (**Supplementary Figure S2**). Overall, these results demonstrated that pharmacological inhibition of AhR suppresses HSV-1 replication *in vitro*, suggesting that AhR acted as a candidate antiviral therapeutic target.

### 3.4 The AhR facilitates HSV-1 replication by promoting the expression of viral genes and viral receptors

**Figure 5:**
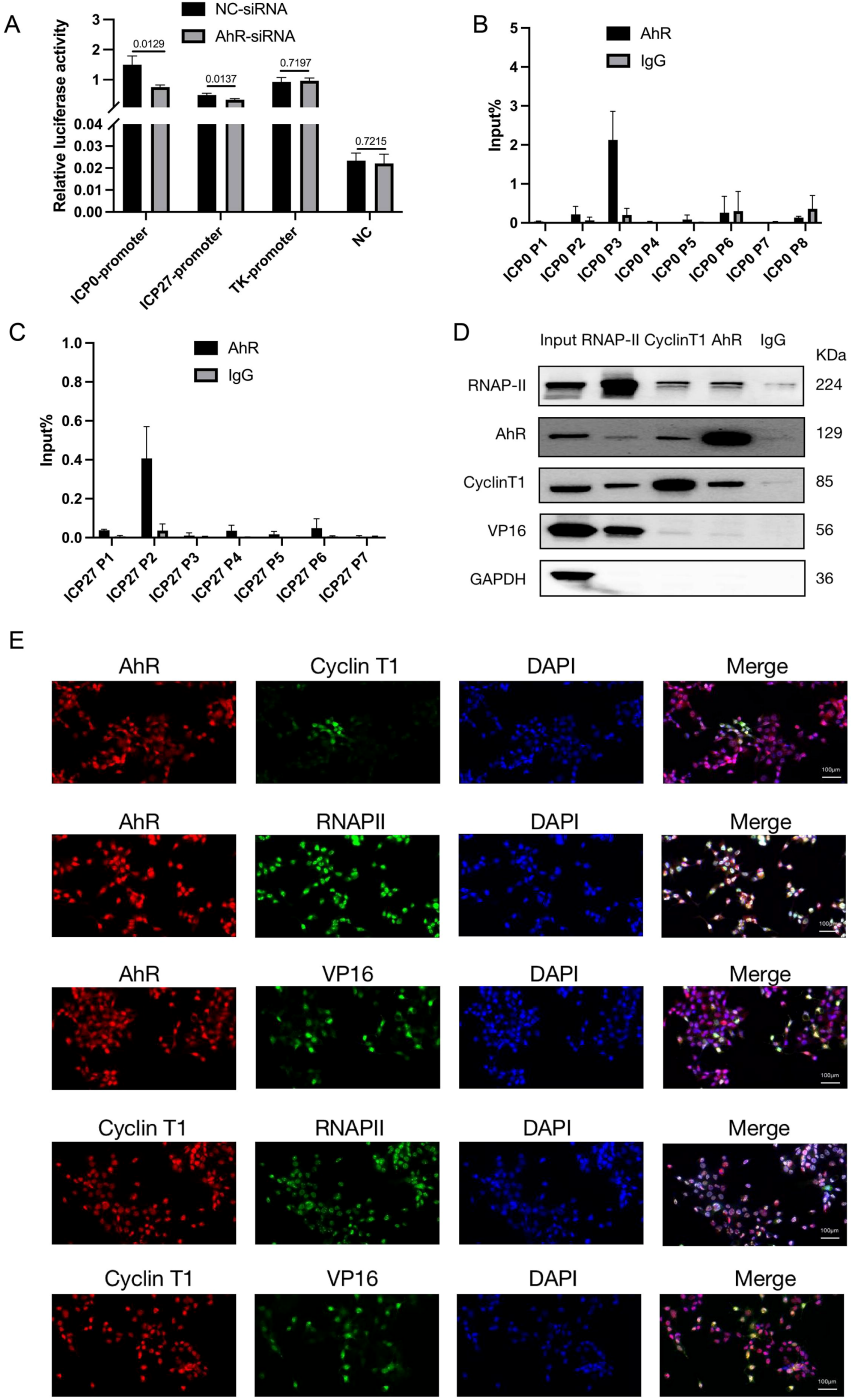
AhR forms transcription complexes with RNAP II, Cyclin T1, and VP16 that bind to viral ICP0 and ICP27 promoters and promote their transcription. **(A)** ICP0, ICP27 and TK promoters of transcription activity analysis. HepG2 cells were transfected with AhR-siRNA or NC-siRNA and luciferase reporter plasmids ICP0-PGL3Enhance, ICP27-PGL3Enhancer, TK-PGL3Enhancer. Meanwhile, PRL-CMV luciferase reporter plasmid was transfected. The cells were infected with HSV-1 (MO1 = 1) 48 hours after transfection, and the samples were collected 4 hours later and analyzed using a dual luciferase reporter assay. **(B)** AhR binds to ICP0 at the P3 segment of ICP0. We infected HepG2 cells with HSV-1 (MOI=10) and collected cell lysate 12 hours later. ChIP and qRT-PCR were used to detect AhR-binding fragments in the ICP0 promoter. The relative content is calculated with the fragment content in Input being 100%. **(C)** AhR binds to ICP27 at the P2 segment of ICP27. HepG2 cells were infected with HSV-1 (MOI=10), and cell lysate was collected 12 hours later. ChIP and qRT-PCR detected ahR-binding fragments in the ICP27 promoter. The relative content is calculated with the fragment content in Input being 100%. **(D)** Co-IP assay of AhR and RNAP II, cyclin T1, and VP16 proteins. HepG2 cells were infected with HSV-1 (MOI=10), and samples were collected 6 hours later for Co-IP. Western blot was used to detect the binding of RNAP-II, AhR, cyclinT1, VP16, and GAPDH proteins. **(E)** Immunofluorescence to determine the intracellular co-localization of AhR, RNAP II, cyclin T1, and VP16 proteins. HepG2 cells were infected with HSV-1 (MOI=1), and samples were collected 12 hours later for immunofluorescence. The co-localization of AhR, RNAP II, cyclin T1, and VP16 proteins was observed under confocal fluorescence microscopy. The scale bar is 100 μm.

In HSV-1 infection, the IE gene is first activated by the viral VP16 protein, which then activates the E and L genes, promoting viral protein expression and viral replication (Nichol et al., 1996). To investigate the mechanism of AhR regulating HSV-1 replication, we firstly used a dual luciferase reporter assay to examine whether AhR directly regulates the transcriptional activity of viral *ICP0, ICP27*, and *TK* gene promoters, which are those genes required for appropriate expression of early and late viral gene products. We generated three luciferase reporter constructs by inserting the promoter fragments of ICP0, ICP27 and TK into pGL3-enhancer vectors (**Supplementary Figure S3**). Afterward, HepG2 cells were co-transfected with the constructed PGL3-Enhancer plasmids and AhR-siRNA. The PRL-CMV luciferase vector was used as an internal control. At 48h after transfection, the cells were infected with HSV-1. At 4hpi, the cells were collected to measure the luciferase of *Fireflies* and *Renilla*. As showed in **Figure 5A**, the relative luciferase activities of ICP0 and ICP27 promoters in the cells transfected with AhR-siRNA were significantly lower than those cells transfected with the NC-siRNA. And for TK promoter, there was no significant difference in relative luciferase activity between AhR-siRNA group and NC-siRNA. These results suggested that AhR regulated the transcriptional activity of ICP0 and ICP27 promoters, but it has no regulatory effect on TK promoter. Notably, these results are consistent with the regulatory effect of AhR on viral proteins, as we previously described.

As a transcription factor, AhR participates in the process of transcriptional initiation by forming a transcriptional initiation complex with RNA polymerase II in gene promoter region. To determine the AhR binding sites in the ICP0 and ICP27 promoters, we designed sets of primer pairs that recognized the upstream and downstream transcriptional start sites (TSSs) of these genes (**Supplementary Table S3**), and performed a chromatin immunoprecipitation (ChIP) assay with anti-AhR antibody or anti-IgG antibody in HSV-1-infected HepG2 cells.

As showed in **Figure 5B**, the Input% value of the P3 segment of the ICP0 promoter precipitated by the AhR antibody was significantly higher than that of other segments, suggesting that AhR was mainly bound to the P3 fragment of the ICP0 promoter. Similarly, the Input% value of the P2 segment of the ICP27 promoter precipitated by the AhR antibody was significantly higher than that of other segments (**Figure 5C**), indicating that AhR was mainly bound to the P2 fragment of the ICP27 promoter. The results showed that AhR was enriched near the TSSs of ICP0 gene and ICP27 gene, indicating that AhR enhances viral gene transcription by binding to gene promoters.

Next, the co-immunoprecipitation (Co-IP) assay was performed to determine which proteins were recruited by AhR to regulate the transcription of the *ICP0* and *ICP27* genes. It has been reported that activated AhR can recruit positive transcription elongation factor cyclin T1 and RNA polymerase II (RNAP II) to promote gene transcription (Zhou et al., 2019), and VP16 can be combined with Cyclin T1 to recruit RNAP II to promote HSV-1 gene transcription (Kurosu and Peterlin, 2004). Therefore, we analyzed the interactions of AhR with Cyclin T1, VP16, and RNAPII in HepG2 cells infected with HSV-1 by the Co-IP and Western blot. The results showed that the AhR antibody can pull down the RNAPII, Cyclin T1 and VP16 proteins. Similarly, the RNAPII antibody can pull down the AhR, Cyclin T1 and VP16 proteins, and the Cyclin T1 antibody can also pull down the AhR, RNAPII and VP16 proteins (**Figure 5D**). These results demonstrated that AhR can bind with Cyclin T1. And Cyclin T1 can combine with RNAPII. VP16 can also combine with RNAPII. Thus, a transcriptional complex was formed with RNAPII as the core, including VP16, Cyclin T1, and AhR as components.

**Figure 6:**
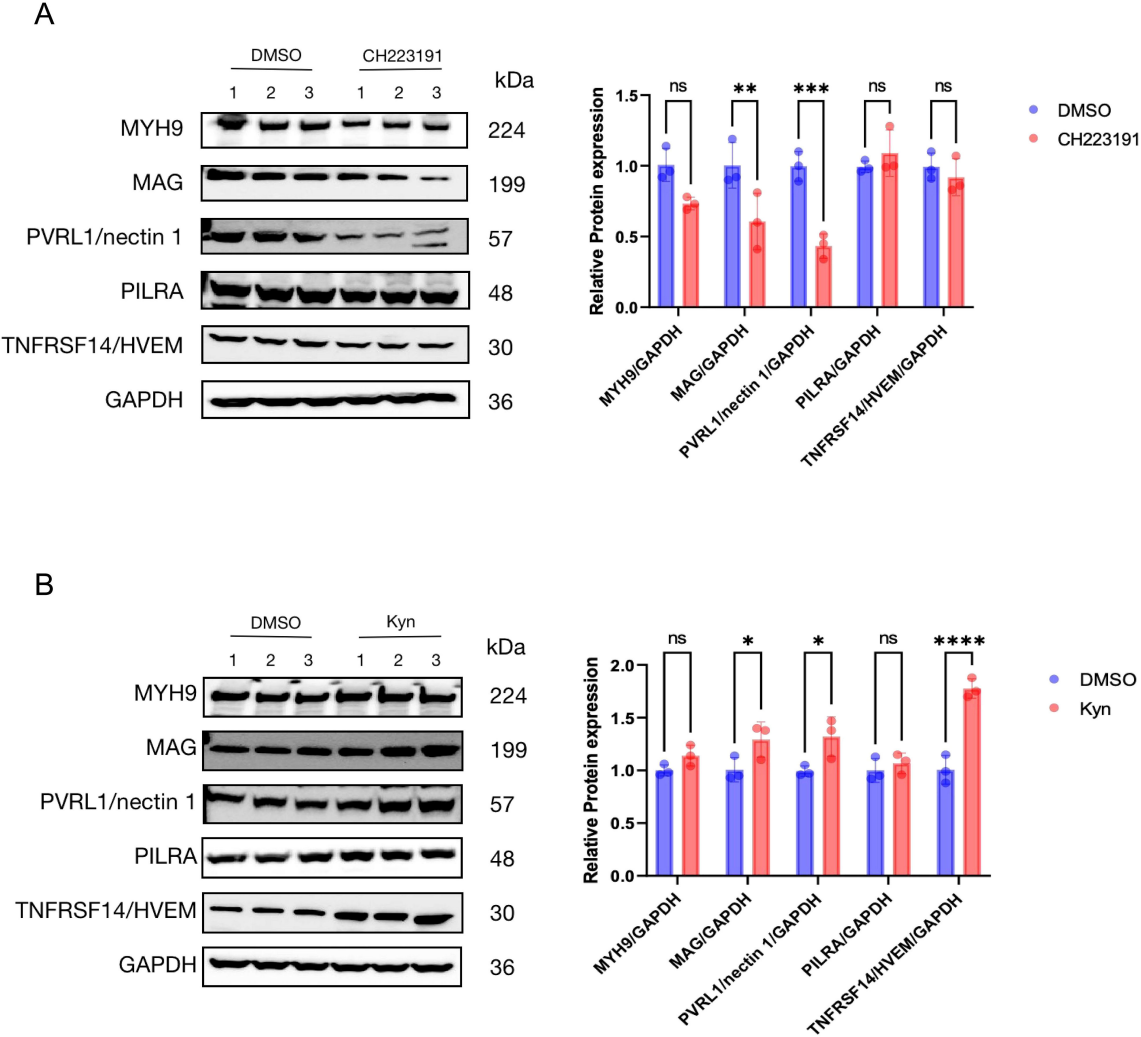
AhR promotes the expression of multiple HSV-1 receptors **(A)** Huh7 cells were treated with CH223191 (30 μM) for 24h and **(B)** Huh7 cells were treated with kyn (400 μM) for 24h. Western blot was then used to detect the expression of viral receptors. Protein ratios of the indicated proteins/GAPDH were analyzed with ImageJ software, and statistical analysis was performed with data from three independent experiments (right panel). In the picture,ns represents no significant difference. A P value less than 0.05 is considered significant. *, **, ***, and **** represent p≤0.05, p≤0.01, p≤0.001, and p≤0.0001.

Furthermore, we tested the co-localization of AhR with Cyclin T1, VP16, and RNAP II in HepG2 cells infected with HSV-1 by the immunofluorescence assay. The fluorescence of AhR and Cyclin T1, VP16, and RNAP II in cells were completely merged (**Figure 5E**), suggesting an obvious co-ocalization of AhR and Cyclin T1, VP16, and RNAP II. Taken together, these results suggested that AhR activation enhanced the transcriptional activity of the ICP0 and ICP27 promoters through the co-localization of AhR with viral gene promoter, Cyclin T1, VP16, and RNAPII.Our previous study has confirmed that AhR facilitated ACE2 transcription to promote SARS-CoV-2 infection (Shi et al., 2023), so we hypothesized that AhR could act as a transcription factor to promotesthe expression of viral entry receptor, including HSV-1 gD and gB receptors (gD receptors: TNFRSF14/HVEM and PVRL1/nectin 1; gB receptors: PILRA, MAG and MYH9). To test this hypothesis, we analyzed the expression changes of the five viral entry receptors in Huh7 cells treated with the AhR antagonist CH223191 or AhR natural agonist Kyn. The results showed that the expression of the HSV-1 gD and gB receptors was significantly reduced in the CH223191-treated cells compared with the DMSO-treated cells (**Figure 6A**). On the contrary, the expression of the HSV-1 gD and gB receptors was significantly increased in Kyn-treated cells compared with DMSO-treated cells (**Figure 6B**). Overall, these results suggested that AhR facilitates HSV-1 entry receptors expression.

## 4 Discussion

**Figure 7:**
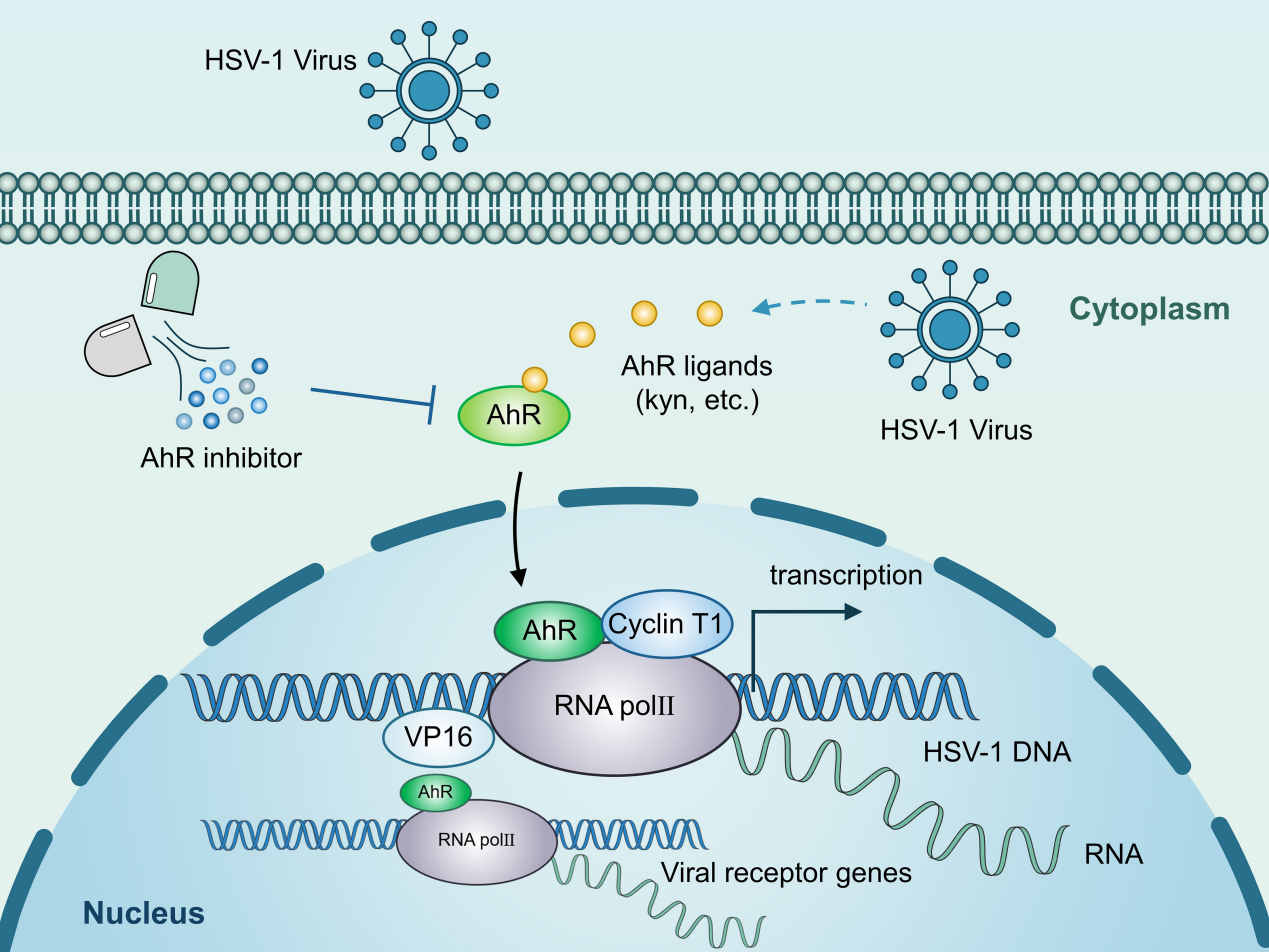
Schematic illustration of the underlying mechanism by which AhR modulates HSV-1 and viral receptor transcription. AhR activation stimulated by HSV-1 increases AhR nuclear translocation. Accumulated AhR is recruited to the promoters of viral IE genes with RNAP II, cyclin T1, and VP16 to facilitate the transcription of these genes. Also, AhR promotes viral gD and gB receptor expression to facilitate HSV-1 infection.

The infection and pathogenesis of HSV-1 have not yet been fully elucidated, especially the key cellular and molecular events of viral lytic infection. Our present study found that HSV-1 infection activates AhR signaling, which then promotes viral replication by regulating viral gene expression at the transcriptional levels. Specifically, AhR is recruited to the promoters of *ICP0* and *ICP27* with RNAP II, cyclin T1, and VP16 to initialize the transcription of these genes. Also, AhR promotes viral gD and gB receptor expression to facilitate HSV-1 infection (**Figure 7**). We also discovered that HSV-1 infection activates the AhR signaling in an interferon-dependent manner. Interferons have a key role in innate immunity, conferring antiviral status to cells in the event of viral infection (Goubau et al., 2013; Jiang et al., 2018; Honda et al., 2006). The stimulation of upregulation of IDO1 by interferon in immune system cells was previously reported (Werner et al., 1987; Carlin et al., 1989). The IDO1 is an intracellular non-secretory enzyme that promotes the metabolism of tryptophan to kynurenine, and kynurenine produced by tryptophan metabolism acts as a ligand to activate AhR, allowing it to enter the nucleus and regulate the expression of downstream genes. HSV-1 infection stimulates interferon production. Yet, this study found that HSV-1 infected cells produce less interferon than positive controls, which may be related to HSV-1 infection of cells antagonizing the host’s innate immune defense system.

AhR has been reported to be associated with the infection of many viruses, including Zika virus (Marim et al., 2021), human cytomegalovirus (Bodaghi et al., 1999), SARS-CoV-2 virus (Shi et al., 2023), measles virus 40, and HIV (Zhou et al., 2019). This study showed that HSV-1 could not replicate efficiently in AhR^−/−^ HepG2 cells, and activating AhR could promote viral replication, while its inhibition of AhR could reverse this process, which is consistent with a recent study (Shi et al., 2023). However, the previous study does not clarify the molecular mechanism of AhR in regulating viral replication. Thus, our study elucidated the molecular mechanisms underlying AhR regulation of *HSV-1* viral gene transcription and host cell receptor expression. Because AhR is a nuclear transcription factor, we hypothesized that AhR can enter the nucleus and directly act on the expression of HSV-1’s genome regulatory gene. Although not reported in DNA viruses, this mode of action has been found in RNA viruses, where AhR binds directly to the 5′-LTR of the HIV-1 virus at the molecular level, recruiting positive transcription factors into the viral promoter region to facilitate viral transcription and infection (Zhou et al., 2019). Our study found that the mechanism of AhR promoting HSV-1 replication at the level of viral gene transcription is very similar to that of the HIV-1 virus, and that AhR translocation into the nucleus may very likely result in formation of a transcriptional complex in the nucleus consisting of cyclin T1, VP16, and RNAP II binding to viral ICP0 and ICP27 promoters and facilitating viral gene transcription transcription (or transcription from the viral genome), promoting HSV-1 replication.

AhR facilitates viral receptor expression, including HSV-1 gD and gB receptors, thereby potentially promoting HSV-1 intercellular transmission. There are two modes of enveloped virus transmission, namely cell-free release (CFR) and cell-to-cell-spread (CCS) (Zhong et al., 2013; Sattentau, 2008; Cifuentes-Munoz et al., 2020). In CFR, the progeny virus is released into the extracellular environment and can be spread or infect new target cells; in CCS, the progeny virus is delivered directly to cell-cell junctions (e.g., tight junctions and adhesion junctions) (Rice, 2021). CCS may be a more advantageous transmission mode because it typically occurs in a spatial environment unaffected by the extracellular environment, thus protecting the virus from neutralizing antibodies and other soluble immune effector factors. Although HSV-1 can be transmitted using CCS and CFR, CCS is often chosen (Johnson and Baines, 2011). This pattern of propagation has been recorded in epithelial cells (Dingwell et al., 1995) and neurons (Dingwell et al., 1994) and between neurons and epithelial cells (Howard et al., 2014). Although the exact mechanism of HSV-1 CCS is unknown, the virus at cell-cell junctions can interact more effectively with viral receptors and improve transmission efficiency (Rice, 2021; Johnson and Huber, 2002). In this study, we found that the expression of multiple viral receptors was upregulated after AhR activation, which was undoubtedly more conducive to the spread of the virus. Overall, our study reveals two mechanisms that promote the transcription and replication of HSV-1 after AhR activation, indicating that the mode of action of viruses and hosts is diverse, and even for the same factor AhR, there may be more than one mechanism of action.

Herein, we emphasized that AhR is a pleiotropic transcription factor involved in immune regulation, xenobiotic metabolism, and maintenance of epithelial and neuronal homeostasis (Stockinger et al., 2014; Rothhammer and Quintana, 2019). Chronic or systemic AhR inhibition could disrupt these processes, potentially leading to immune dysregulation or metabolic imbalances. We discuss existing studies on AhR antagonists (e.g., CH223191) in preclinical models, noting that short-term, localized administration may minimize off-target effects. For example (Vogel et al., 2013)reported transient AhR inhibition in acute viral infections without significant toxicity in murine models. We propose that future antiviral strategies could employ tissue-specific delivery systems (e.g., nanoparticle-based targeting) or transient dosing regimens to mitigate risks to uninfected cells. We contextualize the safety profile of AhR antagonists relative to nucleoside analogs like acyclovir, which are associated with nephrotoxicity and myelosuppression. Unlike these drugs, AhR inhibitors may avoid direct interference with host DNA replication (Piret and Boivin, 2016), potentially reducing cytotoxicity (Gutierrez et al., 2024; Kim et al., 2006; Wu et al., 2024). In summary, this study revealed the molecular mechanisms underlying AhR regulation of HSV-1 immediate early gene transcription and viral receptor expression. These findings demonstrated that AhR is a proviral host factor for HSV-1, and thus may be used as a promising host-directed antiviral target.

## Data Availability

The datasets presented in this study can be found in online repositories. The names of the repository/repositories and accession number(s) can be found in the article/**Supplementary Material**.

## Supplementary material

Supplementary Figure 1 Enhanced AhR signaling promotes HSV-1 replication. The Vero cells were pretreated with I3S at different concentrations (100μM, 300μM, and 500μM) for 24h. After infection with HSV-1 (MOI=1) for 24h, the cells were analyzed using immunofluorescence. Viral protein was observed under a fluorescence microscope. The green fluorescence represents the viral protein, and the scale is 100μm.

Supplementary Figure 2 Cell viability analysis of CH223191 and I3S. **(A, C, E, G)** Untreated Vero **(A)**, Huh7 **(C)**, SK-N-SH **(E)**, and HepG2 cells **(G)** were treated with CH223191 at different concentrations. Cell viability was detected 24 hours later. **(B, D, F, H)** Vero **(B)**, Huh7 **(D)**, SK-N-SH **(F)** and HepG2 cells **(H)** were treated with I3S at different concentrations. Cell viability was detected 24 hours later. Cells treated with DMSO as a solvent or blank control represented untreated cells.

Supplementary Figure 3 Construction of the plasmids PGL3Enhancer-ICP0, PGL3Enhancer-ICP27, and PGL3Enhancer-TK. **(A)** PGL3Enhancer-ICP0, PGL3Enhancer-ICP27, PGL3Enhancer-TK double enzyme digestion and electrophoresis. M is the DNA Marker DL2000. The number 1 represents the PGL3Enhancer-ICP0 plasmid digested by KpnI and XhoI; the number 2 represents the PGL3Enhancer-ICP27 plasmid digested by KpnI and XhoI, and the number 3 represents the PGL3Enhancer-TK plasmid cleaved by KpnI and XhoI. **(B–D)** are the forward sequencing results of PGL3Enhancer-ICP0, PGL3Enhancer-ICP27, and PGL3Enhancer-TK plasmids.
