# Supplementary figures and images for: Aryl hydrocarbon receptor facilitates HSV-1 lytic infection by enhancing viral gene transcription and receptor expression
Source: Front Cell Infect Microbiol. 2025 Jun 25;15:1548038. doi: 10.3389/fcimb.2025.1548038 (PMC12238220; doi:10.3389/fcimb.2025.1548038)

Fig. S1

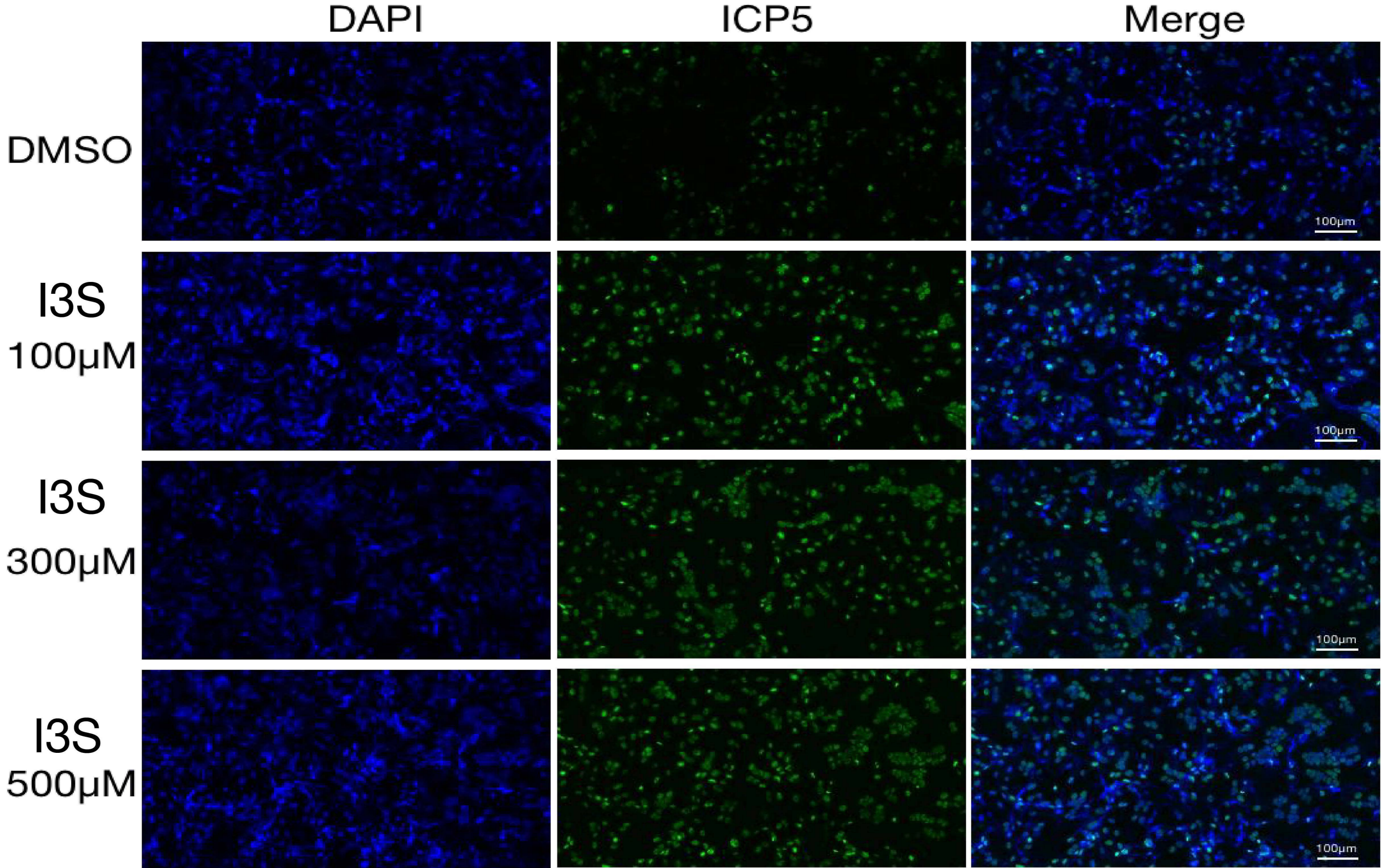

Supplement: Supplementary Figure 1 — Enhanced AhR signaling promotes HSV-1 replication. The Vero cells were pretreated with I3S at different concentrations (100μM, 300μM, and 500μM) for 24h. After infection with HSV-1 (MOI=1) for 24h, the cells were analyzed using immunofluorescence. Viral protein was observed under a fluorescence microscope. The green fluorescence represents the viral protein, and the scale is 100μm. [file DataSheet1.pdf]

Fig.S2

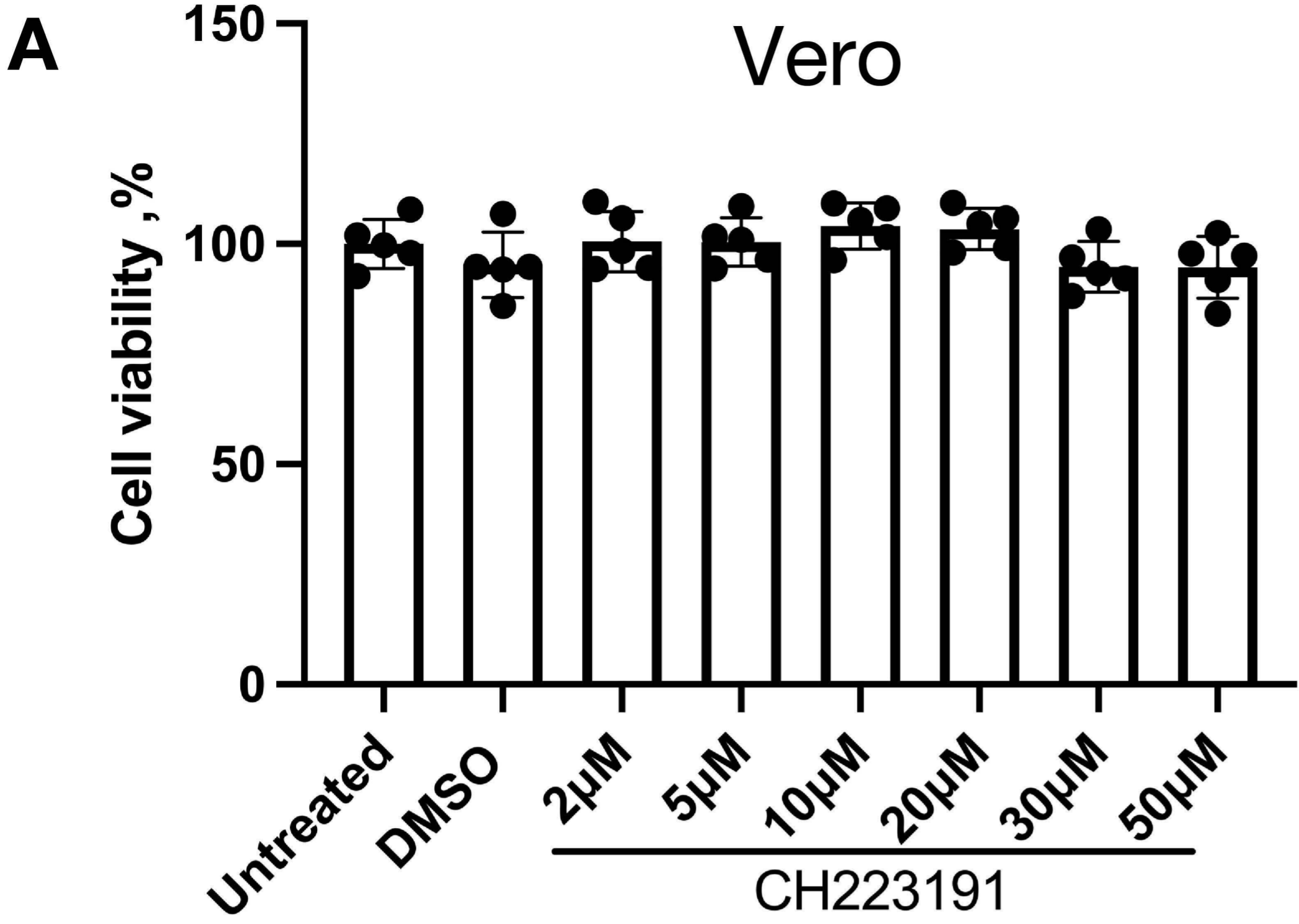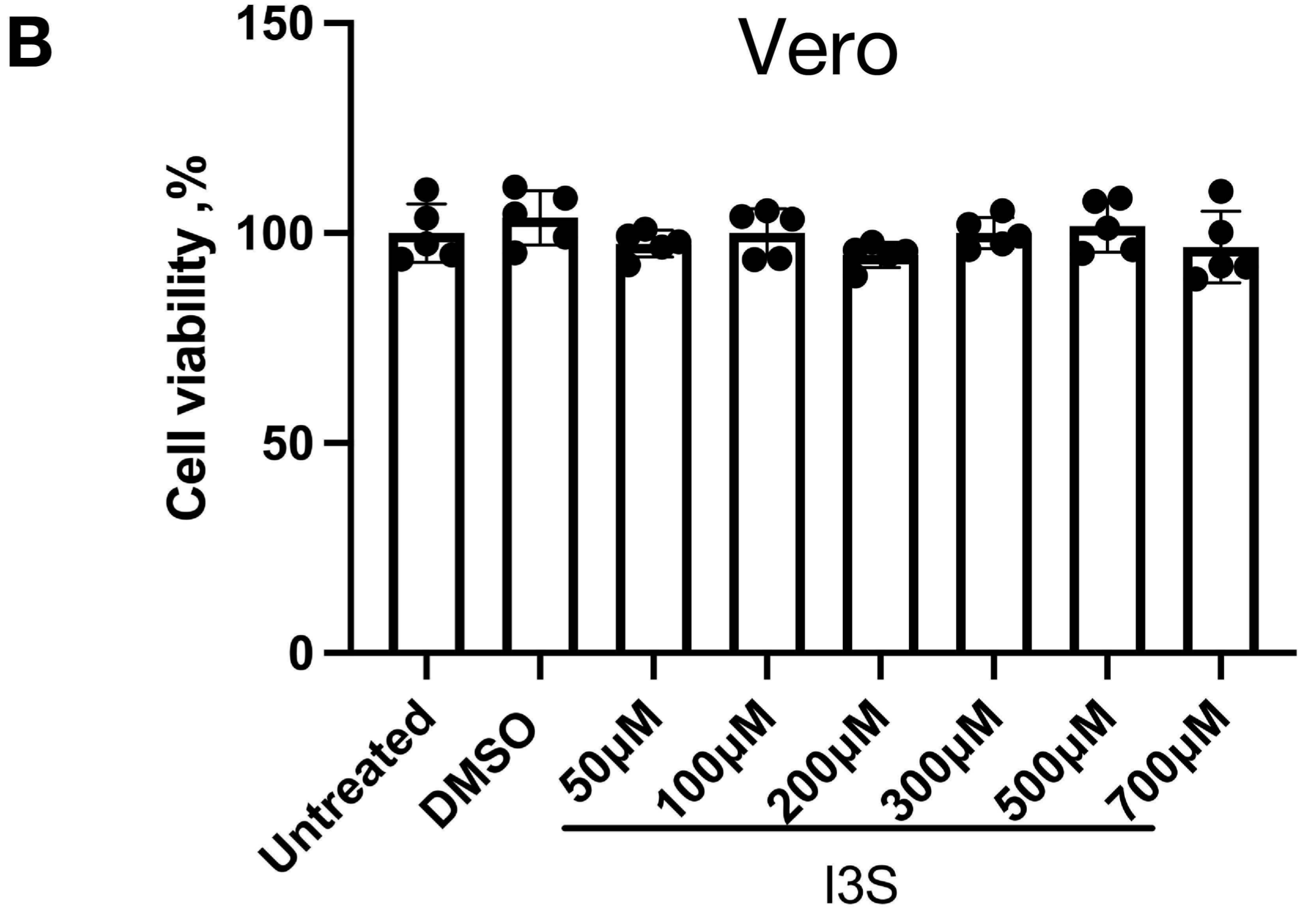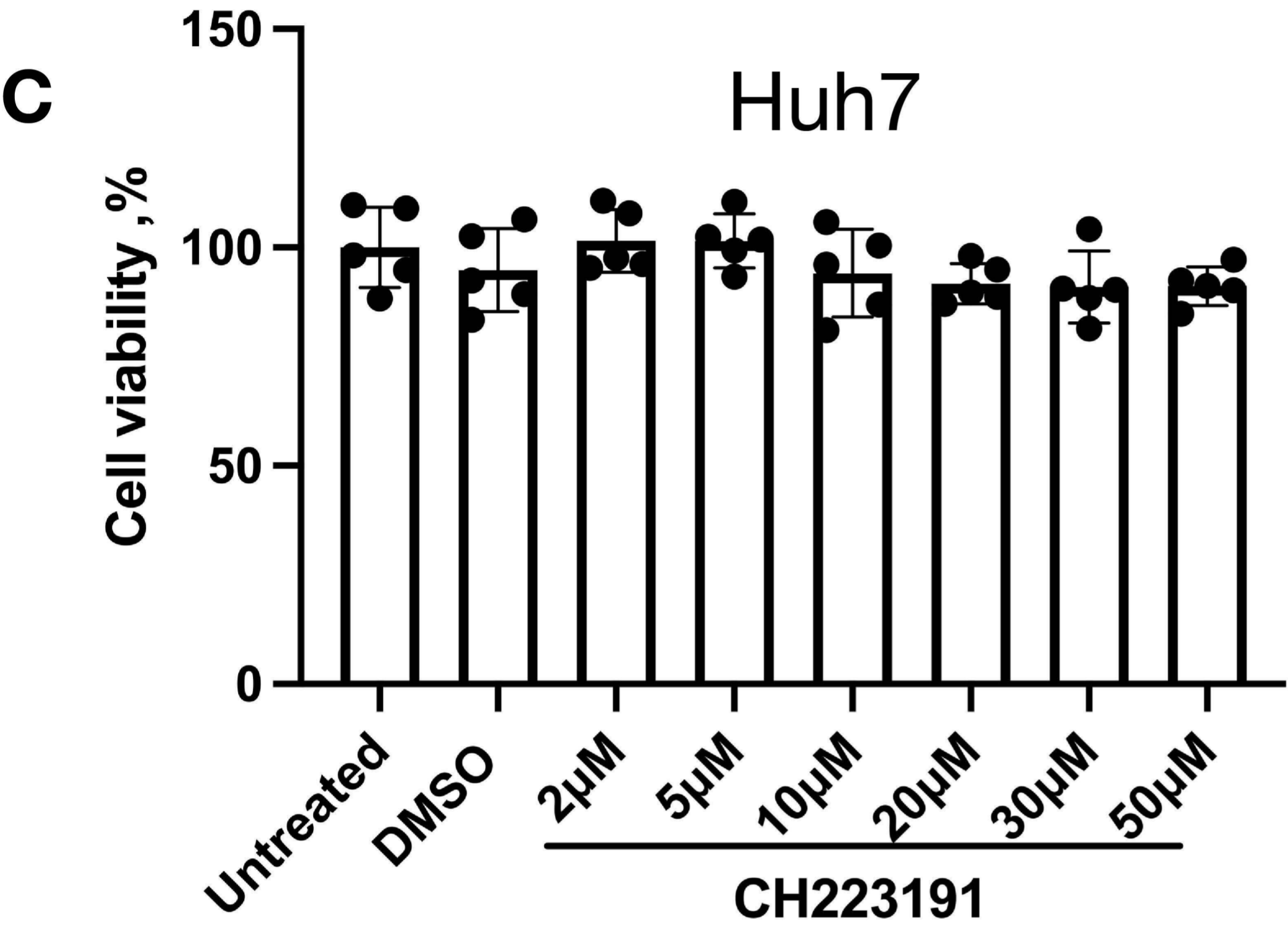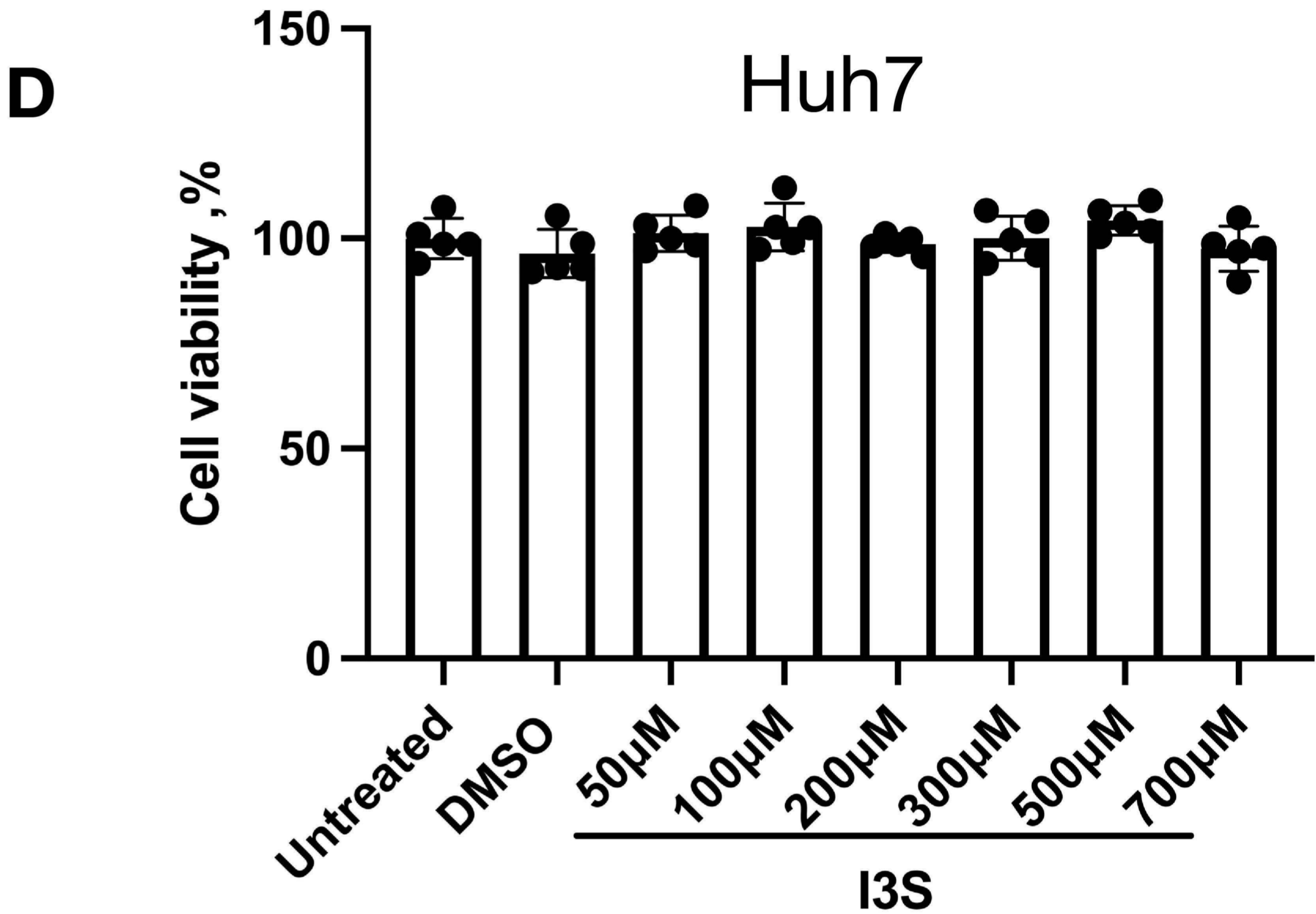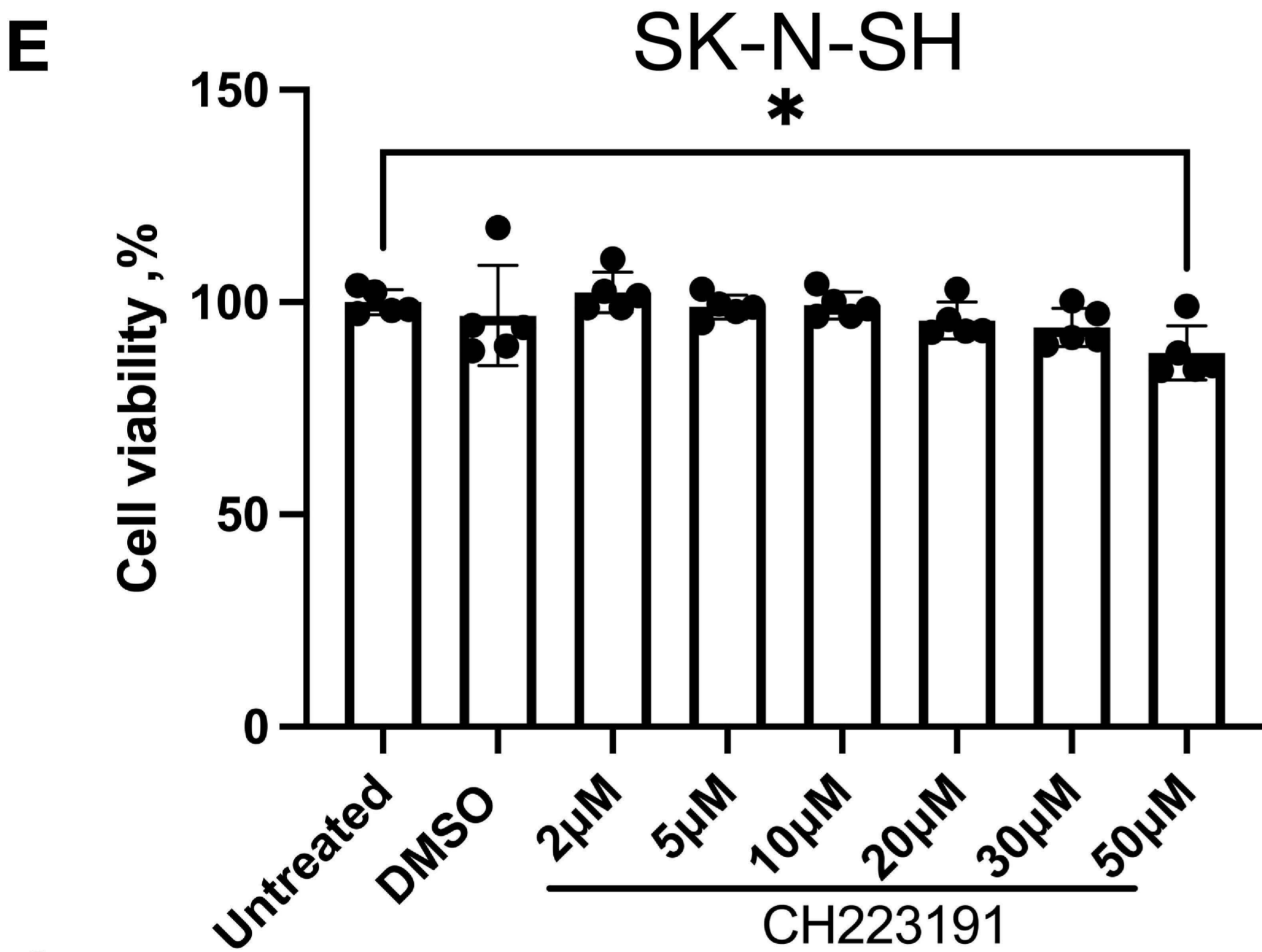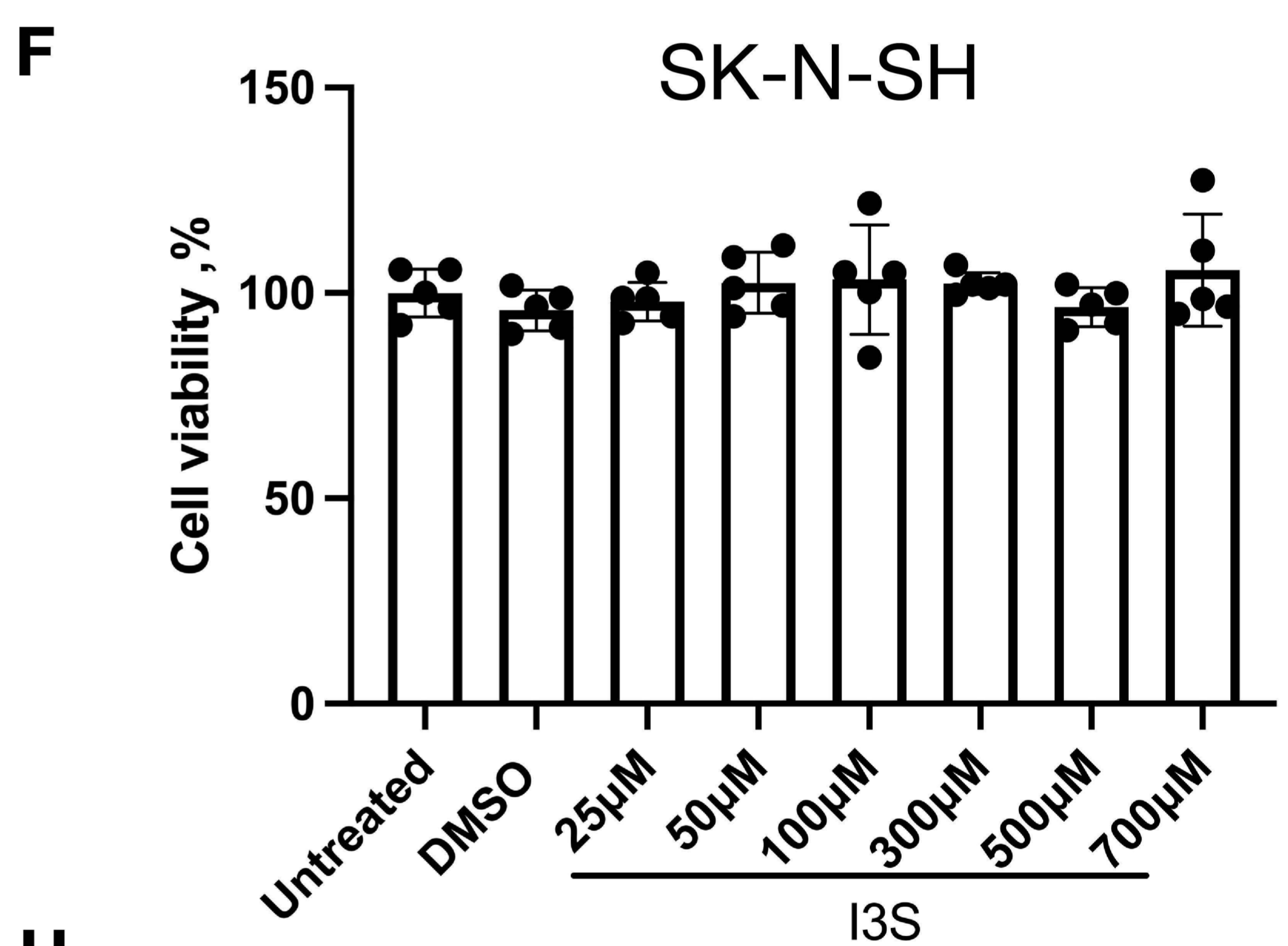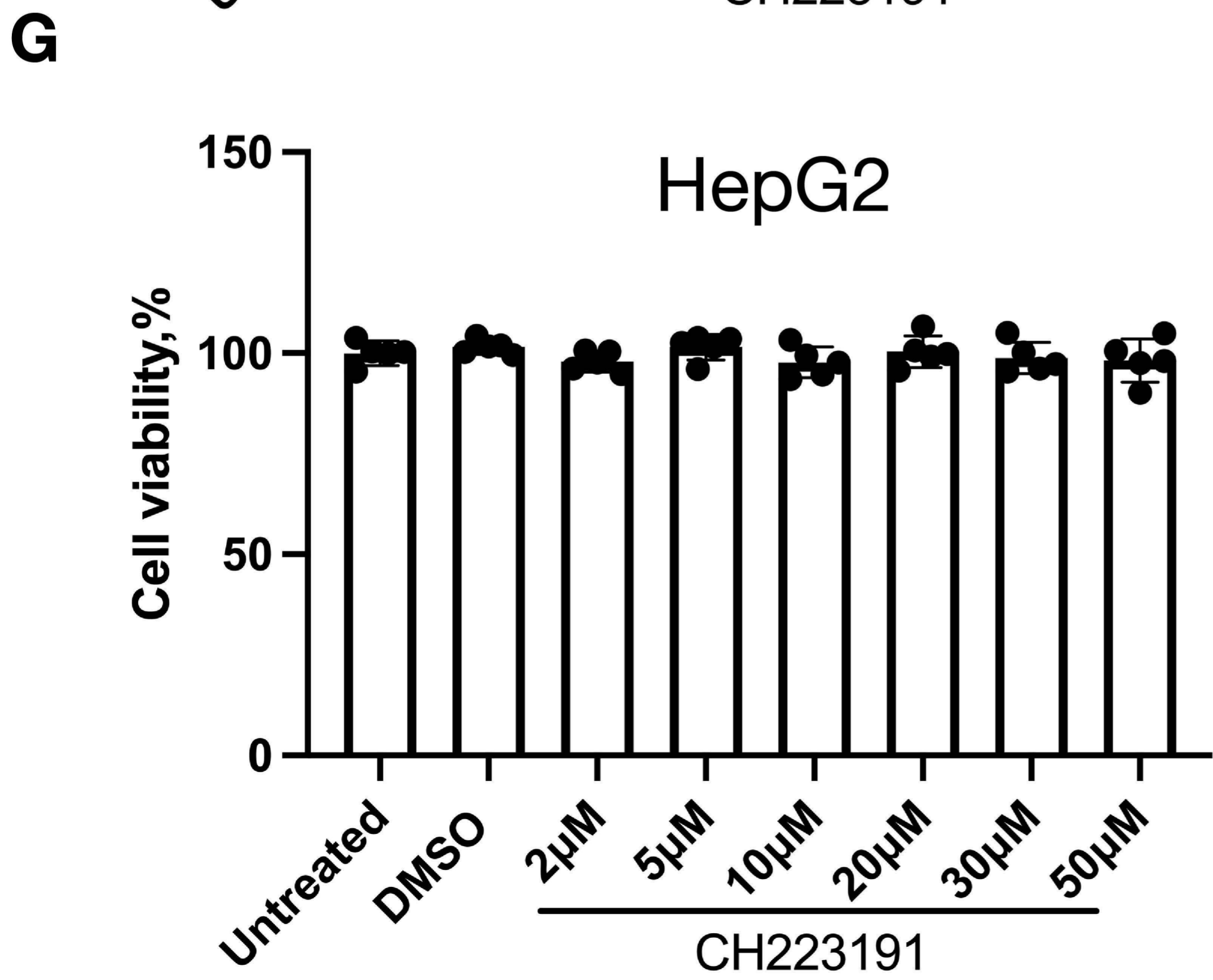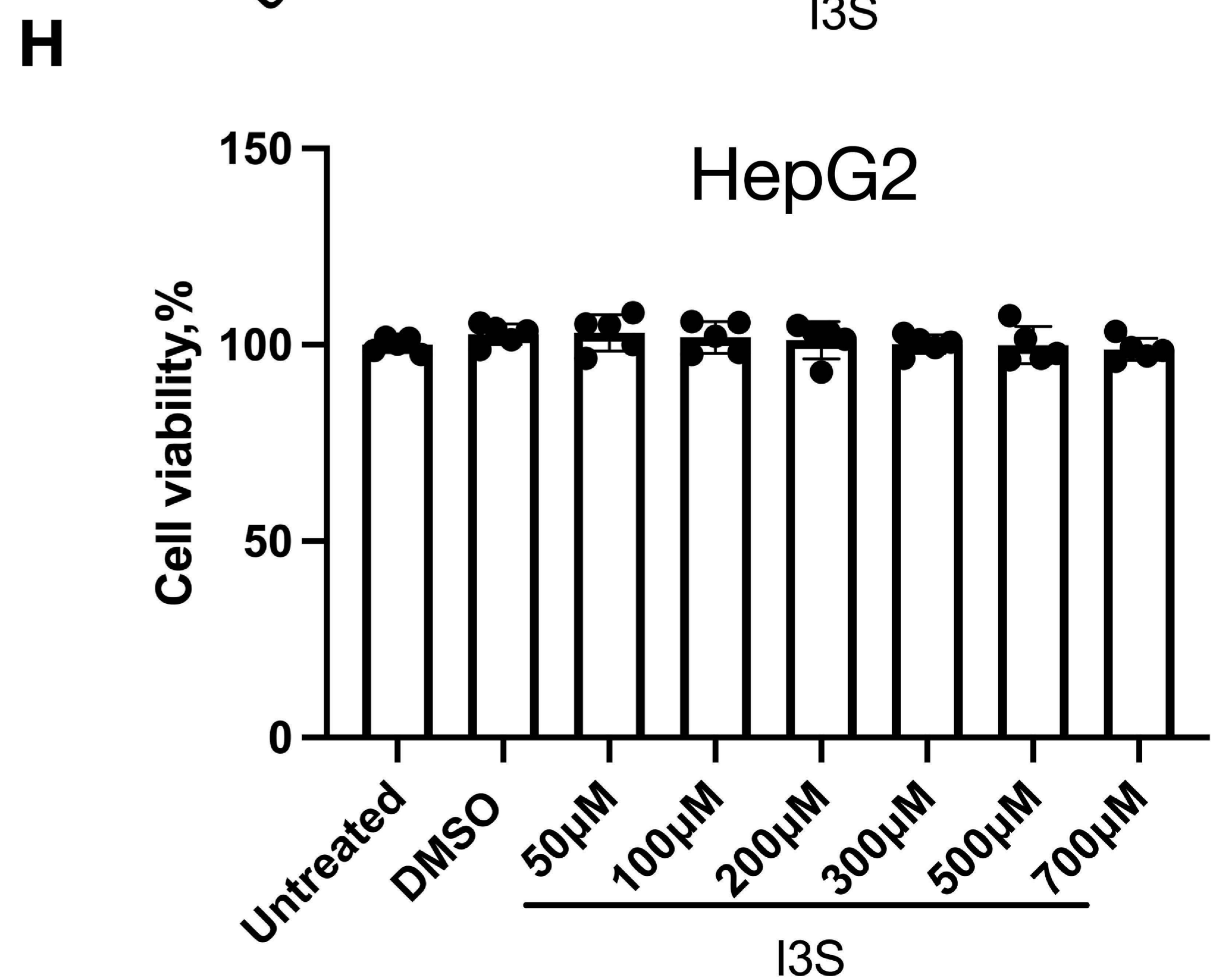

Supplement: Supplementary Figure 2 — Cell viability analysis of CH223191 and I3S. (A, C, E, G) Untreated Vero (A), Huh7 (C), SK-N-SH (E), and HepG2 cells (G) were treated with CH223191 at different concentrations. Cell viability was detected 24 hours later. (B, D, F, H) Vero (B), Huh7 (D), SK-N-SH (F) and HepG2 cells (H) were treated with I3S at different concentrations. Cell viability was detected 24 hours later. Cells treated with DMSO as a solvent or blank control represented untreated cells. [file DataSheet2.pdf]

# Fig.S3

**A**

|   |   |   |   |
|---|---|---|---|
| M | 1 | 2 | 3 |
|---|---|---|---|

bp

2000

1000

750

500

250

100

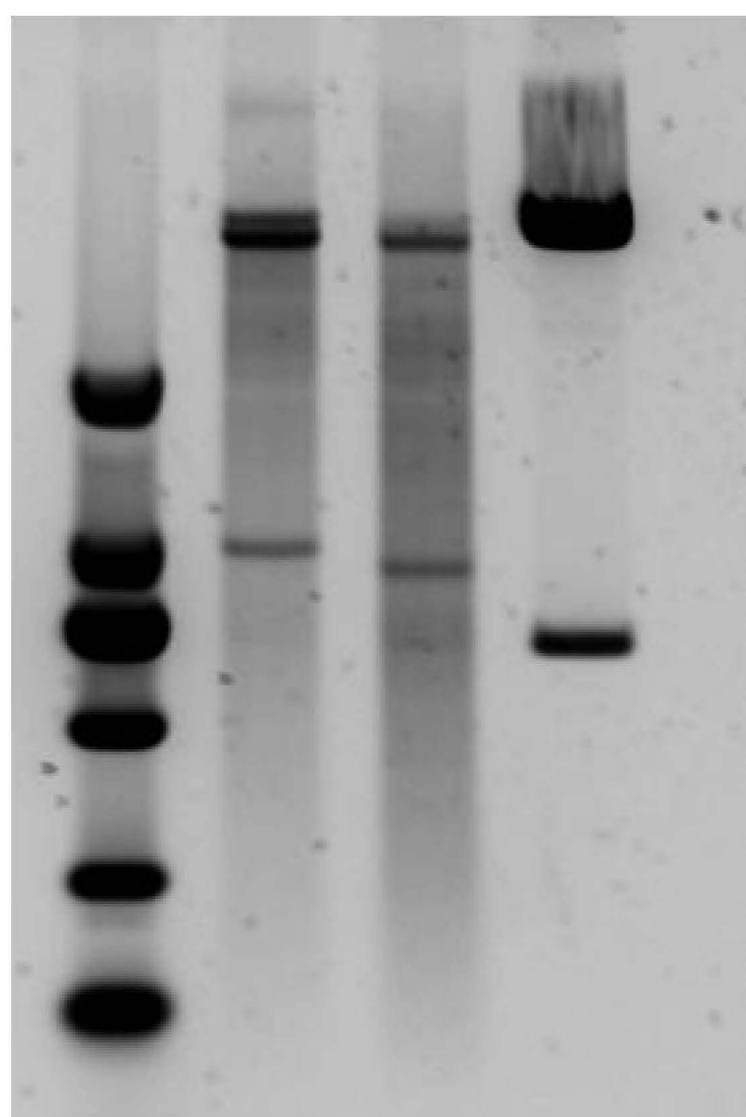

# B

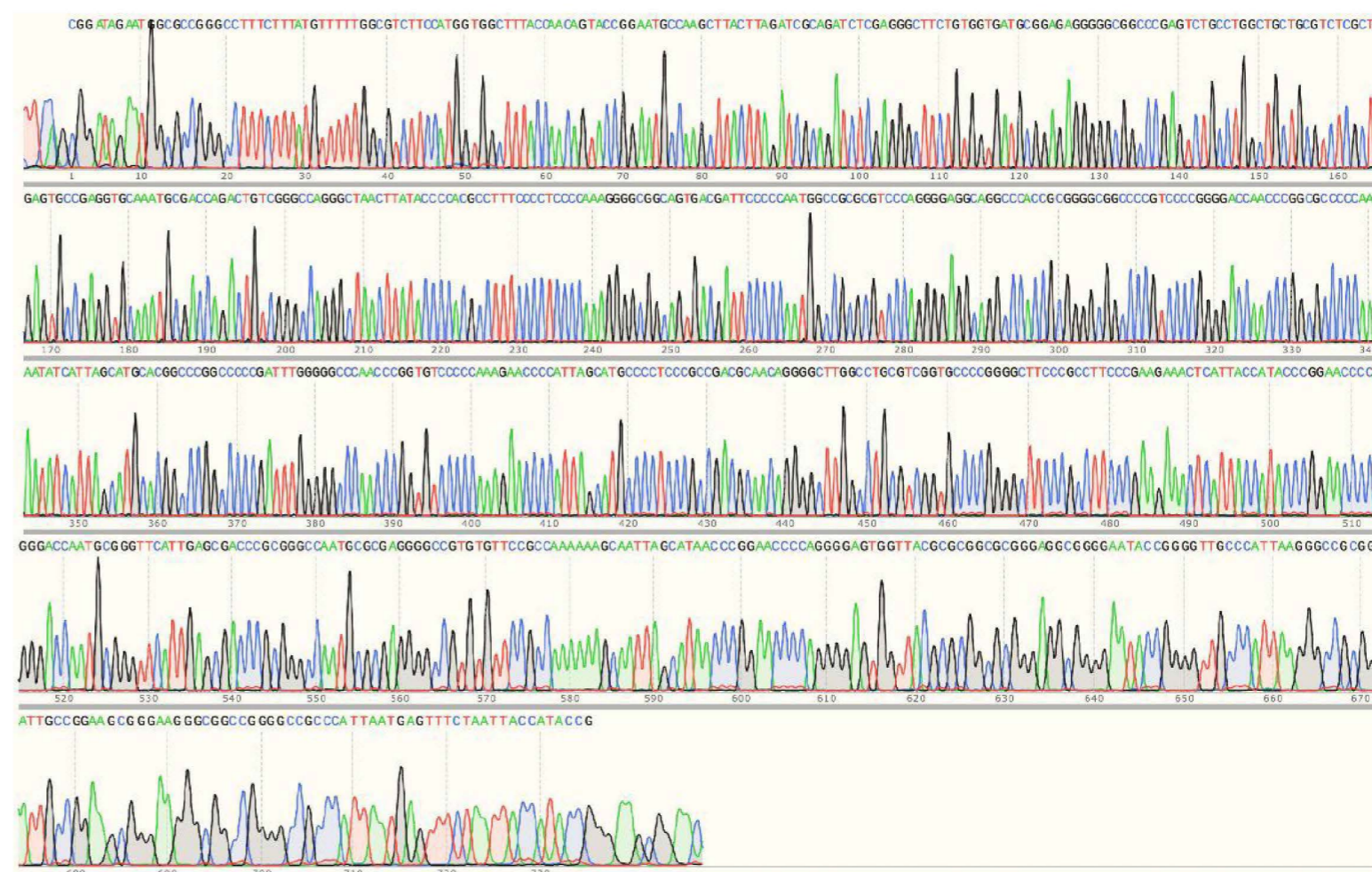

C

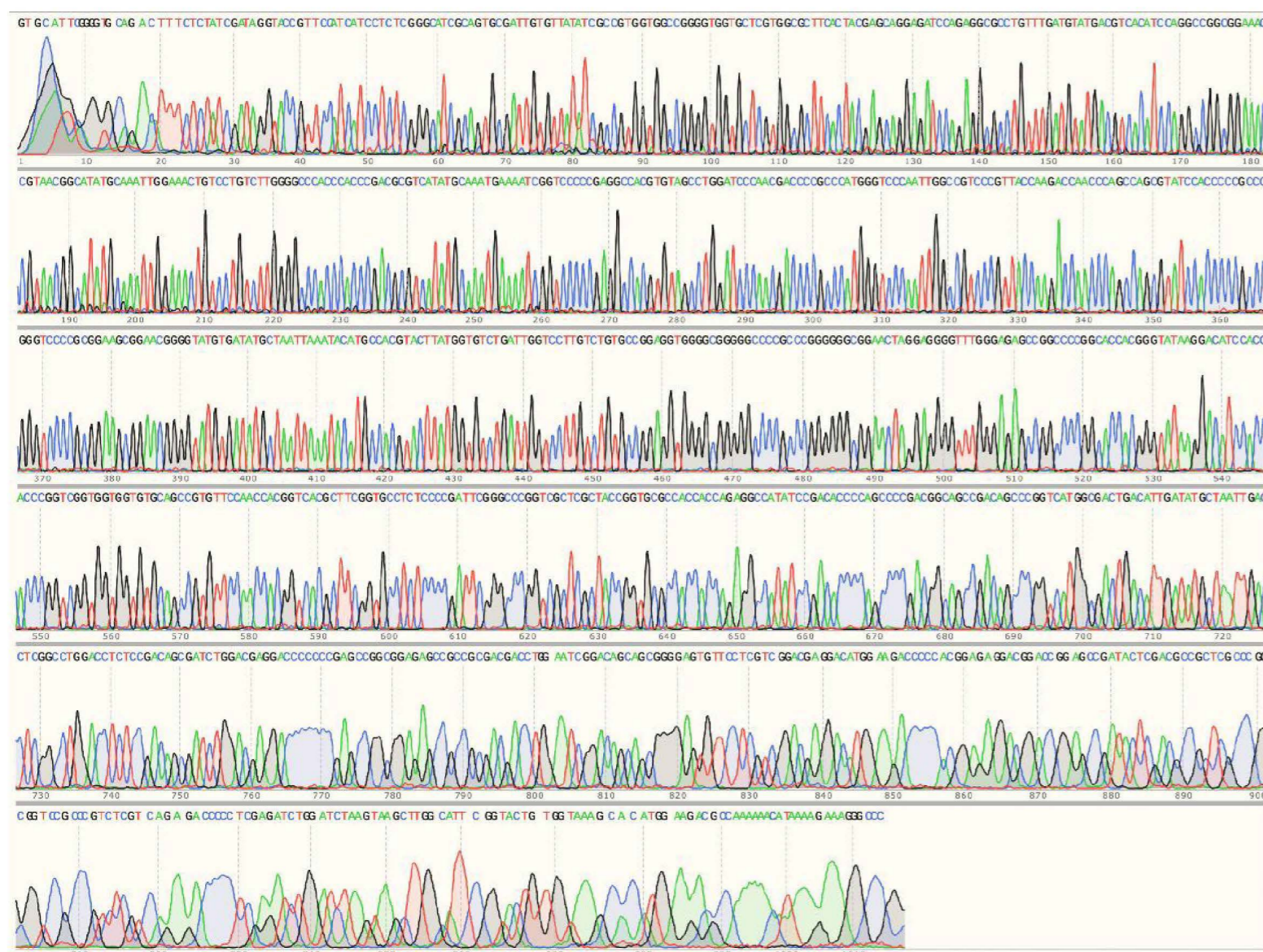

D

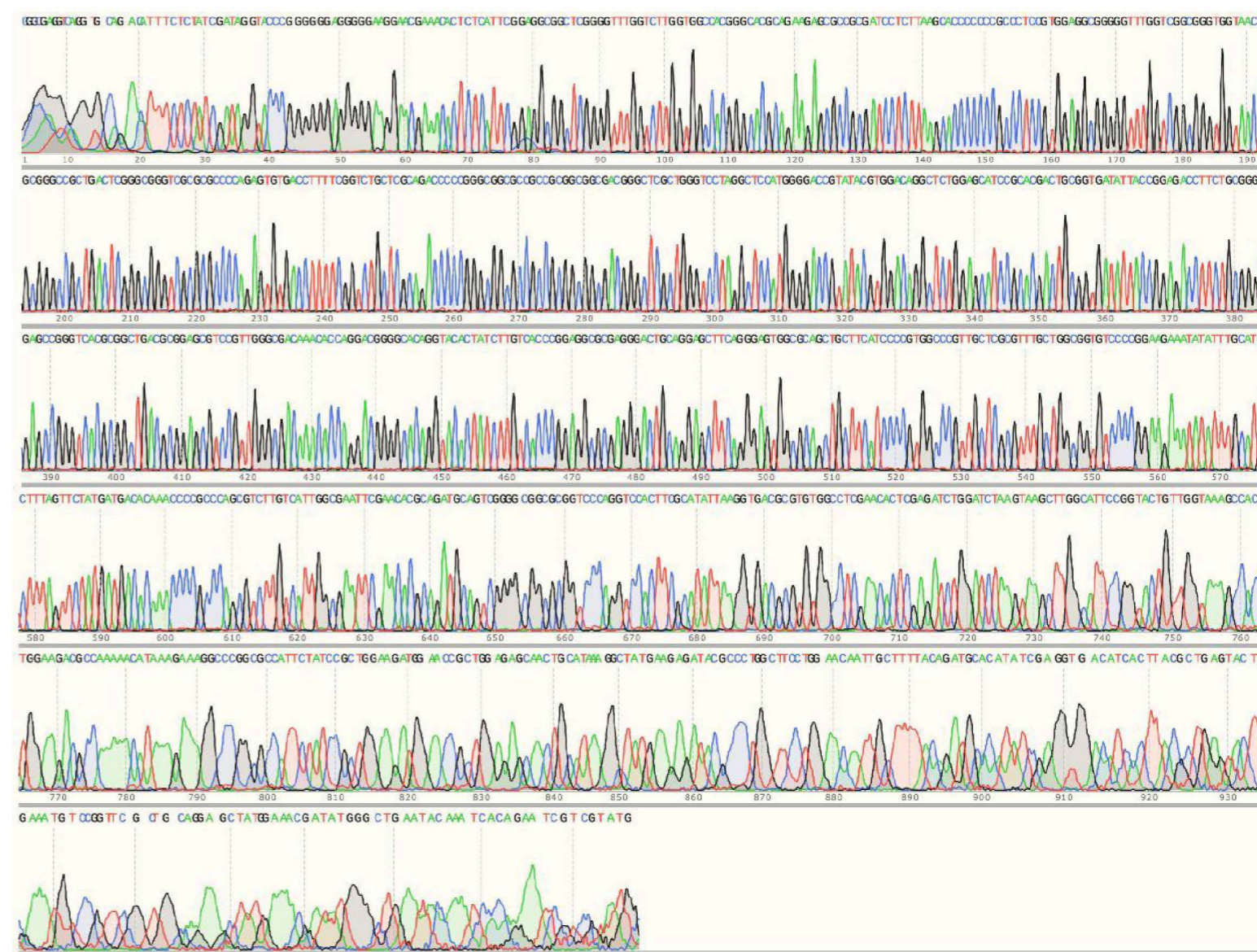

Supplement: Supplementary Figure 3 — Construction of the plasmids PGL3Enhancer-ICP0, PGL3Enhancer-ICP27, and PGL3Enhancer-TK. (A) PGL3Enhancer-ICP0, PGL3Enhancer-ICP27, PGL3Enhancer-TK double enzyme digestion and electrophoresis. M is the DNA Marker DL2000. The number 1 represents the PGL3Enhancer-ICP0 plasmid digested by KpnI and XhoI; the number 2 represents the PGL3Enhancer-ICP27 plasmid digested by KpnI and XhoI, and the number 3 represents the PGL3Enhancer-TK plasmid cleaved by KpnI and XhoI. (B–D) are the forward sequencing results of PGL3Enhancer-ICP0, PGL3Enhancer-ICP27, and PGL3Enhancer-TK plasmids. [file DataSheet3.pdf]
