# Supplementary material for: Aryl hydrocarbon receptor facilitates HSV-1 lytic infection by enhancing viral gene transcription and receptor expression
Source: Front Cell Infect Microbiol. 2025 Jun 25;15:1548038. doi: 10.3389/fcimb.2025.1548038 (PMC12238220; doi:10.3389/fcimb.2025.1548038)

Supplementary Materials for

AhR is a proviral host factor and a candidate target for anti-HSV therapy

Pu Huang[1,4#], Hongli Chen[1,3#], Mengyue Lei[1], Ying Ma[1], Xiaohong Pan[1], Xiaomei Guo[1,3], Jiaxin Xie[1], Jixiong Li[1,3], Jing Sun[1], Yunzhang Hu[1]*, Jiandong Shi[1,2]*

[1]Yunnan Provincial Key Laboratory of Vector-borne Diseases Control and Research, Institute of Medical Biology, Chinese Academy of Medical Sciences and Peking Union Medical College, Kunming, China.

[2]National Kunming High-level Biosafety Primate Research Center, Institute of Medical Biology, Chinese Academy of Medical Sciences and Peking Union Medical College, Kunming, China.

[3]Kunming Medical University, Kunming, China.

[4]Honghe Health Vocational College, Honghe, China.

#These authors contributed equally.

***Corresponding author.**
Email: huyunzhangym@126.com(YZ. H.); shijiandong@imbcams.com.cn (JD.S.)

Supplementary Tables

Table S1. Primers used for reporters construction in this study.

Primer name	Primer sequence (5'-3')
ICP0 F	CGGGGTACCCGGTCTAACGTTACACCCGAGGC
ICP0 R	CCGCTCGAGGGCTTCTGTGGTGATGCGGAGAG
ICP27 F	CGGGGTACCGTTCCATCATCCTCTCGGGCATC
ICP27 R	CCGCTCGAGGGGGTCTTCTGGACGAGACGGG
HSV-1 F	CATCACCGACCCGGAGAGGGAC
HSV-1 R	GGGCCAGGCGCTTGTTGGTGTA
TK F	CGGGGTACCCGGGGGGAGGGGGAAGGAACGAAAC
TK R	CCGCTCGAGTGTTCGAGGCCACACGCGTCACCTT

Table S2. Primer sequences used for qPCR in this study.

Name	Forward primer (5'-3')	Reverse primer (5'-3')
GAPDH	GGAGCGAGATCCCTCCAAAAT	GGCTGTTGTCATACTTCTCATGG
IFNB1	GCTTGGATTCCTACAAAGAAGCA	ATAGATGGTCAATGCGGCGTC
IDO1	GCCAGCTTCGAGAAAGAGTTG	ATCCCAGAACTAGACGTGCAA
TDO2	TCCTCAGGCTATCACTACCTGC	ATCTTCGGTATCCAGTGTCGG
AhR	CAAATCCTTCCAAGCGGCATA	CGCTGAGCCTAAGAACTGAAAG
CYP1A1	ACATGCTGACCCTGGGAAAG	GGTGTGGAGCCAATTCGGAT
CYP1A2	CTGGGCACTTCGACCCTTAC	TCTCATCGCTACTCTCAGGGA
CYP1B1	AAGTTCTTGAGGCACTGCGAA	GGCCGGTACGTTCTCCAAAT
HSV-1	CATCACCGACCCGGAGAGGGAC	GGGCCAGGCGCTTGTTGGTGTA
HSV-1 Probe	FAM-CCGCCGAACTGAGCAGACACCCGCGC-TAMRA	

Table S3. Primer sequences used for ChIP assay in this study.

Name	Forward primer (5'-3')	Reverse primer (5'-3')	Location (Relative to TSS)
ICP0-P1	CCGACAGTCTGGTCGCATTT	GGCTCCATGGGGGTCGTAT	18/183
ICP0-P2	CCATTGGGGGAATCGTCAC	CTTCTGTGGTGATGCGGAG	-52//111
ICP0-P3	GGGCATGCTAATGGGGTTCT	GCAGTGACGATTCCCCCAAT	-205/-31
ICP0-P4	CAATGAACCCGCATTGGTCC	AGAACCCCATTAGCATGCCC	-318/-184
ICP0-P5	CTTAATGGGCAACCCCGGTA	CGCCTTCCCGAAGAAACTCA	-446/-257
ICP0-P6	CTTGTTCCGCTTCCCGGTAT	GAATACCGGGGTTGCCCATT	-532/-424
ICP0-P7	TCTAACGTTACACCCGAGGC	CGTATATATGCGCGGCTCCT	-832/-728
ICP0-P8	AGACAGGCAAGCACTACTCG	TGGAGGTTACCTGGGACTGT	-1653/-1481
ICP27-P1	GACGAGGACATGGAAGACCC	CTGGTTGAGGATCGTTGGGG	139/298
ICP27-P2	CCAGAGGCCATATCCGACAC	CTGTCCGATTCCAGGTCGTC	-51/116
ICP27-P3	CGTCCCGTTACCAAGACCAA	CGGCACAGACAAGGACCAAT	-376/-240
ICP27-P4	CCATCATCCTCTCGGGCATC	GCCCCAAGACAGGACAGTTT	-654/-473
ICP27-P5	TCACCTTCTTGTACCACCGC	AACACCAGGTGGTGATGGTC	-942/-784
ICP27-P6	GCCGATGTATGTTTGGCGTC	GCAGGCGGGTAATTTTCGTG	-1131/-1000
ICP27-P7	TCCGCTGCACCGATGTATTT	CCCGCGATAAGATTGGCGTA	-1460/-1290

Notes: Primer positions are indicated relative to transcription start site (TSS).

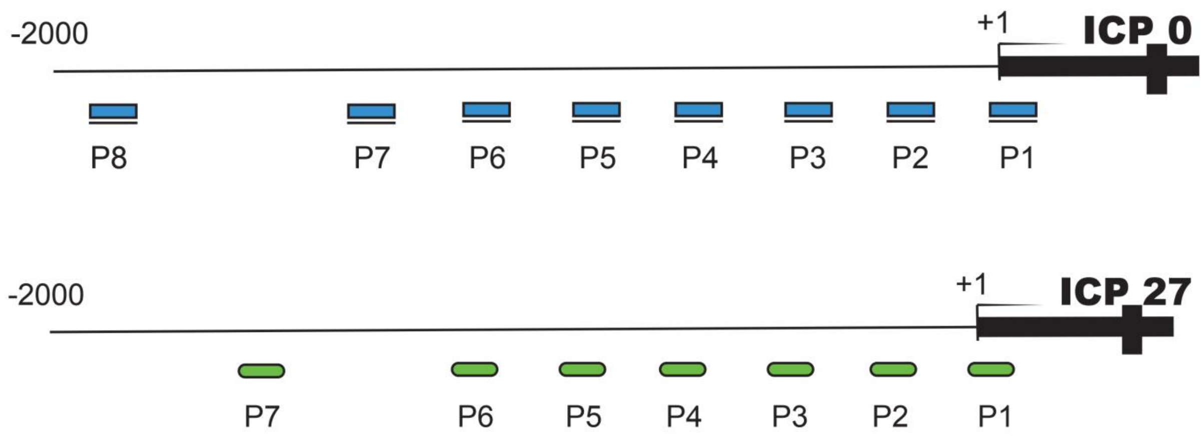

Supplement: Supplementary file 4 [file DataSheet4.pdf]
